# Why are some languages confused for others? Investigating data from the Great Language Game

**DOI:** 10.1371/journal.pone.0165934

**Published:** 2017-04-05

**Authors:** Hedvig Skirgård, Seán G. Roberts, Lars Yencken

**Affiliations:** 1 The Wellsprings of Linguistic Diversity Laureate project, Department of Linguistics, School of Culture, History and Language, College of Asia and the Pacific, Australian National University, Acton, ACT, Australia; 2 Language & Cognition Department, Max Planck Institute for Psycholinguistics, Nijmegen, Gelderland, Netherlands; 3 Independent researcher, Melbourne, Victoria, Australia; Leiden University, NETHERLANDS

## Abstract

In this paper we explore the results of a large-scale online game called ‘the Great Language Game’, in which people listen to an audio speech sample and make a forced-choice guess about the identity of the language from 2 or more alternatives. The data include 15 million guesses from 400 audio recordings of 78 languages. We investigate which languages are confused for which in the game, and if this correlates with the similarities that linguists identify between languages. This includes shared lexical items, similar sound inventories and established historical relationships. Our findings are, as expected, that players are more likely to confuse two languages that are objectively more similar. We also investigate factors that may affect players’ ability to accurately select the target language, such as how many people speak the language, how often the language is mentioned in written materials and the economic power of the target language community. We see that non-linguistic factors affect players’ ability to accurately identify the target. For example, languages with wider ‘global reach’ are more often identified correctly. This suggests that both linguistic and cultural knowledge influence the perception and recognition of languages and their similarity.

## Introduction

Objective studies of similarities and differences of languages allow researchers to reconstruct patterns of historical change in linguistic structures [1], or social changes such as migrations [2, 3]. However, knowledge of linguistic variation is also important in the day-to-day life of non-linguists. Being confronted with, and often confounded by, a different accent or a foreign language is perhaps a universal human experience; we are often in situations where we hear snippets of unfamiliar conversation in a public space or on the radio, and we try to identify what it is we hear. People are sensitive to differences in pronunciation and vocabulary and are quick to link these differences to their knowledge of cultural differences. Our attitudes and the way we change our behaviour when trying to communicate past these differences form part of the basis for language change, but is very hard to study.

In some cases, identifying non-group members by linguistic means may be a matter of life or death, as in the Biblical episode involving the pronunciation of Hebrew *s(h)ibboleth* ‘ear of wheat’ (Judges 12:6). More recent examples include the 20th-century civil war in Sri Lanka where allophonic differences were used to differentiate Tamil and Sinhalese speakers (see [4]). Indeed, linguistic diversity can be driven by the need to differentiate between cultural groups [5] (see also Thurston’s concept of ‘esoterogeny’ [6] and Larsen’s ‘nabo-opposisjon’ [7]). In extreme cases, a biased attitude towards a language can severely affect perception, even to the extent of reducing comprehension [8, 9].

In spite of the importance of perceiving linguistic differences in daily life, there have been few studies exploring the factors which influence people’s ability to recognise the languages of the world and their similarities. This game presents us with a new kind of data that has previously not been available, and offers the chance to investigate the perception of language similarity. In this paper, we analyse the results of an online game played millions of times by a global sample of people, in which the goal was to accurately identify languages based on a 20-second audio clip of speech. Based on the players’ behaviour, we can extract information about which languages they can easily identify, and where they get confused. We use the results of this game to investigate what factors make a language easy to identify accurately, including non-linguistic factors such as economic power and the quality of the audio recordings. We also assess whether languages which are often confused also have some objective linguistic similarity, for example being closely related, geographically close, or having similar sound systems or lexicon.

More specifically, the research questions we address in this paper are:

- Q1: Which languages are confused for which others?
- Q2: Are there any asymmetries of confusion?
- Q3: What factors can predict whether players confuse two languages for each other? Candidates include:
  – geographical closeness;
  – genealogy;
  – similarity of phoneme inventories;
  – lexical similarity.
- Q4: What factors can predict players’ accuracy? Candidates include:
  – acoustic quality of the speech samples;
  – proportion of non-native speakers (L2 speakers);
  – total native speaker (L1) speaker population;
  – linguistic diversity of the main country in which the language is spoken;
  – number of countries the language is spoken in;
  – “language name transparency”—how clear the link is between the language name and the name of the main country;
  – economic power of main country as measured by Gross Domestic Product (GDP);
  – Familiarity with the language, as measured by the frequency of occurrence of the language name in written materials such as the English and Chinese corpora from Google Books.
- Q5: Is players’ performance more accurately predicted by linguistic or non-linguistic factors?
- Q6: Are some phonological cues more important than others in predicting players’ accuracy?

### Identifying languages and relationships between them

Dividing a continuum of related linguistic varieties into discrete languages and families is a difficult task. As Evans & Levinson note, “we are the only species with a communication system which is fundamentally variable at all levels [of linguistic organization]” [10]. The most common methods of separating varieties into languages are based on tests of mutual intelligibility, shared literary tradition and/or shared lexicons [11, 12]. However, speaker/signer communities may have their own perceptions and labelling of group identity (cf [13]). The editors of the Ethnologue recognise that “a language is more often comprised of continua of features that extend across time, geography, and social space. There is growing attention being given to the roles or functions that language varieties play within the linguistic ecology of a region or a speech community” [14]. Language users’ own perception of what are salient contrasts and important categories can often differ significantly from what linguists focus on. Often, categorizations by language users are politically motivated. For example, Norwegian and Swedish are considered different languages by their respective language communities, despite there being large groups of speakers from the two communities who are able to understand each other and a large shared lexicon (cf. [15]).

There are at least three types of relationship between languages: temporal, spatial and social. For example, the gradual differences leading from Middle English to Modern English form a temporal continuum of variation within the same community. Linguistic borrowing due to contact can result in a spatial continuum, as found in the areal patterns of East Asia (e.g. Cantonese, Mandarin, Japanese and Korean in our sample) or Mainland Southeast Asia (Khmer, Burmese, Lao and Thai). Often, languages are related both in time (i.e. genealogy) and in space (i.e. geographical proximity), as in the case of the closely-related Germanic dialects of Belgium, the Netherlands, Germany, Switzerland, Luxembourg and Austria. Languages can also vary on a social scale, this is for example reflected in different language varieties within the same language depending on socio-economic status (cf. the basilect-acrolect continuum in creole languages).

Despite variation along the temporal, spatial, and social dimensions, communication between speakers of quite different varieties is both possible and productive, suggesting that we must possess an effective capacity for perceptually handling variation. In this paper, we take a rare chance to investigate non-linguists’ perception of linguistic variation across the world. We excluded guesses made by players from countries where the target language is an official or de facto official language, because it is less interesting that they are able to accurately identify the target. This means that we are most likely getting data on how outsiders (e.g. non-speakers of the target) perceive language similarity.

### Predictions

There is no research, as far as we are aware, which explores the perception of a wide range of languages by outsiders (individuals who are not familiar with the target languages) and how this compares to linguists’ objective measurements of similarity. There is however relevant work in perceptual dialectology (i.e. dealing with very closely related varieties [16]), such as work on listener identification of regional varieties of English from the United States [17, 18]. Listeners are able to identify regional varieties well above chance, and the perception of similarity differs between listeners from different dialect backgrounds and between listeners with varying knowledge of relevant sociolinguistic cues.

Listeners use differences in speech varieties as cues to social identity (e.g. [19]), leading to the formation of stereotypes about certain speech sounds or ways of speaking (e.g. [20]). These attitudes may, in turn, affect the perception of speech sounds (e.g. [8, 21]).

Another study showed that American children could identify American English and Indian English varieties in a forced choice task, but were less good at distinguishing American English and British English [22]. However, the children did link pictures of familiar cultural items (e.g. houses and clothing) to more familiar varieties. This suggests that perceptual and cultural knowledge of variation emerge at a young age. Indeed, young adults with high-functioning autism (who have good perceptual processing, but poor social processing) can identify dialect varieties, but are less good at linking them with social stereotypes [23], suggesting a possible dissociation between the two skills.

An experimental study of the ability of listeners to distinguish between two foreign languages (German vs. Russian) has been carried out [24]. Participants were exposed to recordings of the same speakers producing different languages. They performed above chance, and their success was predicted by their level of exposure to the variety, with even occasional exposure via media aiding performance. This suggests that listeners are sensitive to differences between languages as well as between dialects.

In this paper we analyse the results of the Great Language Game, a popular online game created by one of the authors of this paper, Lars Yencken. Players listen to a sample of speech and must guess which language it is from a set of alternatives. We explore which factors predict which languages are guessed accurately and what factors predict which languages are confused for each other. We tested four factors for confusion (similar phoneme inventories, geographic proximity, genealogy, and shared lexicon) and nine factors for accuracy (acoustic quality of audio clip, proportion of L2 speakers, total L1 speaker population, linguistic diversity of main country, number of countries the language is spoken in, language name transparency, economic power of main country and frequency of occurrence of the language name in written materials). There are other possible factors one could consider, but these are the ones that we predicted would be interesting, and were able to measure. An example of a factor that would be interesting but hard to measure is mutual intelligibility: linguists commonly divide speech varieties into languages based on mutual intelligibility (and shared lexicon), but no measurement of mutual intelligibility of all the languages in our sample exists.

The most obvious prediction we would make is that players will differentiate languages based on phonological properties. In other words, languages which sound more different may be easier to distinguish from each other. Particular phonological features may be more salient than others (tone or retroflex consonants, for example). Lexical items are also a potential cue to which speakers might pay attention: we predict that the more lexical items are shared between two languages, the more often the languages will be confused for each other. Since phonological features and lexical items diffuse through borrowing and historical descent, players may also find it easier to distinguish between languages that are further apart in space (spoken in different countries) or time (distantly related, or not at all related).

Another obvious factor that the player might rely on is simply knowledge of the target language. This effect can be limited to some extent by excluding responses from countries where the language is official or *de facto*-official (as we do below); but it cannot be entirely eliminated. The probability of exposure to the target language may be related to the number of people who speak it, we consider both L1 and L2 speaker populations.

Similarly, the player may rely on their cultural knowledge: a player might not know a language very well, but might have heard it being spoken (e.g. in films or other media) or know a few facts about it. A player’s exposure or cultural knowledge of a language may depend on the ‘global reach’ of the language, which can be reflected in the population size, the level of industrialisation (as measured by, for example, gross domestic product, mentions in written material, how many countries it is spoken in and proportion of L2-speakers). For example, Mandarin has more speakers than Spanish, but Spanish is an official language in many more countries, which may increase its global reach despite having fewer speakers.

We also considered the linguistic diversity of the target language’s main country, as a proxy for cultural diversity. Countries with a unified cultural ‘brand’ might be more easily recognisable and salient in people’s cultural knowledge than a country with diverse cultures and languages (this should by no means be taken as an endorsement of monolingualism by the authors, we believe that diversity and multilingualism are most important and should be encouraged).

Languages discussed more widely might be better known. We estimate this by looking at the frequency of the names of the languages in the Google Books N-gram corpus of English and Chinese texts from 1800–2000 [25].

Another factor is the quality of the recordings. We predict that clearer, higher-quality recordings will facilitate language recognition and therefore lead to more correct guesses.

Finally, players might use various non-linguistic heuristics. For example, participants might rule out candidate languages that they know, and therefore deduce the correct answer, though this is hard to test given our data. Another heuristic is to choose languages based on geographic or cultural proximity. For example, a player might think that a language sounds like Russian, but then see that Russian is not presented as a candidate answer. However, it may be that Slovak is an alternative, the player reasons that Russia and Slovakia are culturally connected, are in the same part of the world, or form part of the same language family, and so they may choose Slovak despite not knowing what Slovak sounds like. This would predict that confusion between languages is related to the geographic distance between them, but also that players might confuse languages which are geographically close, but quite different in terms of their genealogy, for example Latvian (Baltic, Indo-European) and Estonian (Finnic, Uralic).

This heuristic can be applied to some languages more easily than others. For example, the geographic location of “Scottish Gaelic” is transparent from the name, while this is not the case for “Shona”, which is spoken in Zimbabwe. The name “Kannada” may be entirely misleading for Western players who might link it with “Canada”, when it is actually a Dravidian language spoken in India. Therefore, we consider the transparency between the language name, and the language main country. This is referred to as ‘language name transparency’ and can also be linked to the previously discussed idea of a ‘cultural/national brand’.

Thus, in relation to Q3 above, we predict that two languages are more likely to be confused if they are:

- geographically close to each other;
- closely related historically;
- similar in their phoneme inventories;
- similar in their lexicon.

In relation to Q4, we predict that a language is more likely to be guessed correctly if it is:

- represented in the game with speech samples of high quality;
- spoken as a second language by a large proportion of the total number of speakers;
- spoken by a large population as a native language (L1);
- mainly spoken in a country where most people speak the same language (low linguistic diversity);
- spoken in many countries;
- transparently-named;
- spoken mainly in a country with great economic power;
- often mentioned in Chinese Google Books;
- often mentioned in English Google Books.

Finally, since perception might be linked to cultural knowledge or shaped by linguistic experience, we predict that patterns of confusion will differ between players from different countries.

### Caveats

The data in the current study provides us with a unique opportunity, but also carries with it certain problems. On the one hand, there is almost too much data: there are more questions to be asked than we can possibly cover here. We thus restrict ourselves to analysing data pertaining to languages and countries, and leave more fine-grained analyses, such as at the regional or state level, for future research. On the other hand, there is not enough data. The data on player identity is limited, we only know the location of their IP-addresses. Furthermore, the selection of participants is biased (the game requires a computer and the Internet to play, and was developed and distributed in certain social circles, and requires some knowledge of English). Lastly, the sample of languages in the game is not representative of the world’s languages. We try to control for these imbalances, but can only draw tentative conclusions. We will discuss later in the paper how this kind of game could be changed to better serve linguistic research, and present an alternative—LingQuest.

The work of Charlotte Gooskens and colleagues on mutual intelligibility of European languages demonstrates that speakers’ linguistic knowledge is more complicated than it might seem. For example, speakers’ attitudes towards the native speakers of a language might influence their perception of that language [8]. This is something that we will be aware of as we interpret our results, but that we unfortunately cannot test for in a rigorous manner.

## Materials and methods

### The game

“The Great Language Game” (henceforth GLG; see https://greatlanguagegame.com/) was created by Lars Yencken in 2013. The original intent was to increase public awareness of the linguistic diversity of urban areas of Australia, the United Kingdom and the United States. In this section we present the game, the languages in the game and the factors that we have investigated in relation to players’ ability to accurately identify languages and their confusion.

**Fig 1:**
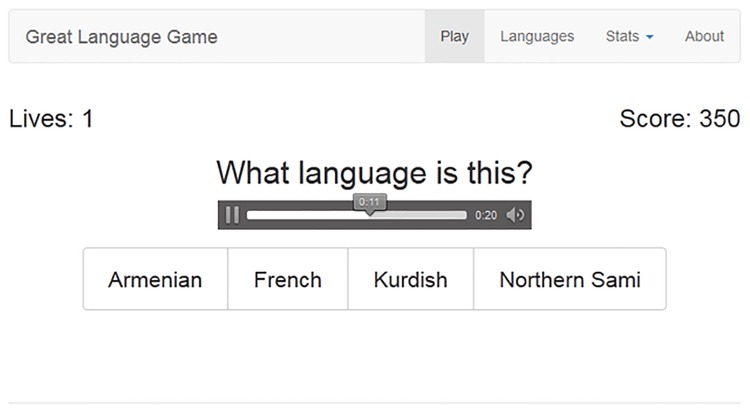
A screen shot from the Great Language Game.

The GLG works as follows: the player listens to a 20-second audio clip of natural speech from one of 78 languages. They must then identify the correct language from a set of alternatives. The alternatives are represented with their common English names. The player can make a choice at any time, there is no time limit or reward for being speedy. They can also play the clip more than once. We do not know how players behaved here. After they make a choice, they are told whether they were correct or incorrect, and are shown the correct answer. In the first round, the player has two alternatives, the correct answer and one distractor. After every three correct answers, the number of distractors increases by 1, up to a maximum of 10 options in total. When the player has made three incorrect choices, the game ends. They are then given some information about the three languages which they guessed incorrectly, as an encouragement to learn more about them. The instructional language of the game is English, but there is very little text that needs to be read in order to play the game. Fig 1 is a screen shot of the game as it appears in a web browser.

In this paper, we make the assumption that the selection of languages as targets in the game is random (see S1 Appendix), although we take into account the number of times the language was offered as a target language. Given an audio clip, the incorrect alternatives (distractors) provided are selected randomly with uniform likelihood. The distractors and the correct alternative are displayed as options in alphabetical order. In practice, the alternatives a player receives may vastly alter the difficulty of a question. For example, the player might be given a set of languages that belong to very different language families (e.g. Polish, Urdu and Tongan), most likely making the task easier. Alternatively, they might be given a set of very closely related languages (e.g. Swedish, Icelandic and Norwegian): a much harder task. They are not awarded any points for selecting a distractor that is in some way more similar to the target than the other distractors are. For example: if the target is Samoan and the alternatives are Samoan, Tongan and Norwegian, you will be equally wrong picking Tongan as you would have been picking Norwegian, despite Tongan being closely related to Samoan.

From here onwards, we describe in more detail the game’s inventory of languages and its player base. Our discussion is based on the GLG’s publicly available 2014-03-02 confusion dataset (see http://lars.yencken.org/datasets/languagegame/ and supporting information S8 Data), and associated audio files from that time (a smartphone clone of GLG was created in 2015 by Isaac Drachman, called “Linguini” which uses many of the same audio clips as GLG, but currently no data has been released for Linguini, so none has been considered in this paper).

### The languages in the game

**Fig 2:**
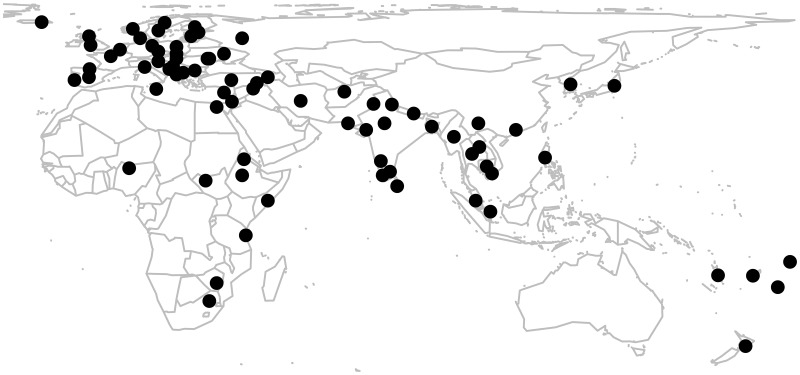
Geographical locations of the languages included in the Great Language Game. The map was created in R using the package *maps* [26]. Coordinates come from Glottolog [12].

At the time of writing, the GLG features 78 languages. For full details on the languages in the game, their genealogy and the rate at which they are guessed accurately in the game, see supporting information S1 Data. Fig 2 shows the geographical location of the languages, with locations taken from Glottolog [12].

The sample of languages in the game is not balanced according to geography or linguistic relatedness. This is evident from the map: Europe is overrepresented and the Americas completely absent. 39 of the 78 languages are from the Indo-European language family. This is because the sample was originally designed to reflect the linguistic diversity of urban areas of Australia, the United Kingdom and the United States, for the purpose of spreading awareness about linguistic diversity in these nations to the general population.

The sample of languages is also dependent on access to speech samples. The speech samples used in the game are mostly drawn from large radio broadcasters which broadcast shows and news in non-majority languages of their respective countries. The majority of the speech samples came from Australia’s Special Broadcasting Service (SBS), Voice Of America (VOA), and British Broadcasting Corporation (BBC). SBS, VOA and BBC broadcast radio and TV in languages spoken by significant populations in Australia, the United States and the United Kingdom respectively. Other smaller sources of speech samples for the game are The Hindu, Phonemica (China), Deutsche Welle, Telugu One, NHK Japan, ORF Eins (Austria), SR (Sweden), CBC (Canada), RFI (France), and the Pacific and Regional Archive for Digital Sources in Endangered Cultures (PARADISEC). PARADISEC is a archive for digital conservation of endangered languages and cultures, with a focus on the Pacific region. At this time, the GLG does not represent the full set of languages available from these broadcasters (English and Kreyòl are for example not present despite being covered by VOA), nor all the languages of PARADISEC.

This, in combination with the fact that the aim of the game is to spread awareness about linguistic diversity in certain specific areas in Western countries, results in under-representation of indigenous languages of other areas, such as the Americas (0 languages, “Americas” here stands for both South America and North America. As a side note it is interesting to observe that despite VOA being located in the United States they do not broadcast news in any indigenous language of the Americas, with the exception of Kreyòl. Kreyòl is a French-lexified creole) and Africa (8 languages) and over-representation of others, such as Europe (33) and Asia (31).

Audio clips come from randomly chosen 20 second segments of broadcasts, identified as being free from excessive noise, music or code-switching (mixing of multiple languages). Most clips are of an informal nature, and may include variable numbers of speakers, laughter, back-channelling and overlapping dialogue. Due to their different sources, clips also vary in audio quality. In the case of telephone interviews, audio quality also varies between speakers within the same clip. Elicited audio recordings created in a controlled lab environment might have less variation in terms of acoustic quality, types of voices, speed etc. This make them more standardised and easy to compare to each other, more suitable for a scientific controlled experiment. However, the clips used in this game have the advantage of being more similar to actual natural discourse, hence more faithful to the actual experiences of people perceiving languages out in the world.

For the purposes of comparing the results of the data from the game to other databases we have linked each language in the game to its appropriate code in the ISO 639-3 set of language codes. The ISO 639-3 is an international standard of language codes used by many linguists and applied in many cross-linguistic databases. In a few cases there are not clear one-to-one mappings; in such events the so called macro language-codes of the ISO 639-2 have been used instead (this concerns Albanian which has been marked as [sqi], Arabic [ara], Dinka [din], Kurdish [kur], Latvian [lav] and Yiddish [yid]). The issue of language names, language classification and ISO-sets is very complicated; for more on this topic see [27] and [28]. In our set some of these complications are made particularly evident by the languages “Bosnian” [bos], “Serbian” [srp] and “Croatian” [hrv] which all are seen as different languages by the Ethnologue and Glottolog, but are perceived as one language by some other linguists and national censuses. We acknowledge these problems, solving them is however beyond the scope of this study.

The issue of language names and codes also becomes a concern when we compare our data with other databases. We compared the confusion data from the game with the similarities and differences between the languages’ phoneme inventories. Data on the phoneme inventories of the languages were taken from the Phonetics Information Base and Lexicon-database (PHOIBLE) [29], which is itself a compilation of information from other data bases. PHOIBLE also employs ISO 639-3, but unfortunately not all languages of the GLG sample had a direct correspondence in PHOIBLE. In the case of the macro-languages of 639-2 we used the sub-variety of 639-3 that was present in PHOIBLE. In a very restricted set of cases we used a neighbouring language variety when there was not a clear mapping; this concerns only Malay ([zlm] in GLG, which was linked to [zsm] in PHOIBLE), Saami [sme −> sma] and Khmer [khm −> kxm]. In other words, for these three languages we did not compare the exact same varieties across our GLG sample and the sample of PHOIBLE. We did however have access to a very closely related variety and we believe that this will have little, if any, effect on the results.

Phoneme inventories for 60 of the languages included in the GLG were found in the PHOIBLE database. PHOIBLE includes data from the SPA (Stanford Phonology Archive), UPSID (UCLA Phonological Segment Inventory Database) and other collections. For many of our languages, there was more than one analysis of the inventory available in PHOIBLE. The different databases that make up PHOIBLE and provide different analyses of the same language differ in their design, rendering direct comparisons over them inappropriate. Languages were only compared if there existed inventories for each of the languages from came from the same source (UPSID was only compared with UPSID, SPA only with SPA). In total there were 31 languages for which we could measure the distance between each pair in this manner. For each language pair, the distance between them was calculated as 1 minus the proportion of phonemes in common. Tone segments were excluded in all comparisons since they were not represented in UPSID.

### The players

The only information about the players which the game elicits is their IP-address, which we use to infer what country they are playing from (the IP addresses were made anonymous in the publicly available data). We make the assumption that the player identifies with this country in some manner (though some may be visiting, travelling, tunnelling their traffic through other countries via VPN or similar technology, etc.). The data includes responses from an estimated 767,000 unique IP addresses (Google Analytics estimates 964,000 unique user identities, meaning that probably many different people play on the same computer). Players can play multiple times, but for simplicity, we assume that most players are casual players who do not play often enough to learn from playing only.

Furthermore, we assume that most players have a knowledge of English, at least sufficiently to find the game in the first place and understand the instructions. The players are also most likely computer-literate and share an interest in languages, or are interested in games of this kind where one measures knowledge of the world and/or linguistics and competes against friends. The game has been extensively shared on certain blogs, forums and social media, where we expect there to be a large number of language enthusiasts/nerds. The players of the GLG come from all over the world, but certain areas are overrepresented. Table 1 displays number of guesses (trials) from each continent.

**Table 1:** Locations of guesses by continent based on IP-address. Note that while there are no indigenous people of Antarctica, there are in fact residents there, varying from 1,100 to 4,400 during the year [30].

Continent of IP-address	Number of guesses
Europe	7,963,630
North America	5,980,767
Asia	841,609
Pacific	364,390
South America	356,390
Africa	74,032
Antarctica	11
Total	15,580,829

### Measuring confusion between languages

The raw data produced by the game is a list of player responses, along with information on the options available to the player in each trial, as well as the country of origin of the IP-address. We use this data to calculate the probability of one language being confused for another, in the following way: given a target language in the speech sample, and a number of alternative language names to choose from, a player selects one language as their guess. From a large sample of games, we know the conditional probability of guessing a particular language G, given that the player actually heard a particular target language T—that is, the chance of a player confusing T for G. This is the proportion of times a player actually guessed G when hearing T compared to the number of times they could have chosen G when hearing T.

$$\text{P}\left(\text{Confuse T for G}\right)=\frac{\text{Number of times a player chooses G given target T}}{\text{Number of times a player could have chosen G given target T}}$$

Note that the correct target language always appears as a candidate guess. In all calculations reported, we excluded guesses made by players from countries where the target language is an official or de facto official language of the country (de facto official languages were included because of cases such as English not being an official language of the UK nor the US). For example, guesses made by players playing from an IP-address in Sweden are excluded when the target language is Finnish (Finnish being an official minority language of Sweden). All languages of the game have been confused for all others at least once.

In addition, the probability of confusion between languages was used to create a distance matrix, where each cell is the inverse probability of one language being confused for another. Languages that are often confused receive a low distance score, while languages that are rarely confused receive a high distance score. When methods required symmetric distance matrices, we used the mean of the probability of A being confused for B and B being confused for A.

Mantel tests were used to explore correlations between these distance matrices. Mantel tests assess the probability of a given correlation between matrices by comparing the actual correlation strength with the correlations when one matrix is permuted (we used 10,000 permutations). While there are methodological problems with Mantel tests [31, 32], they are a useful way of assessing pairwise correlations. Mantel tests were calculated using the R package *ecodist* [33].

We also used Neighbor-Nets to visualize the confusion of languages and phonological similarity. The Neighbor-Net methods require special properties of the distance matrix, such as the triangle inequality. This is defined as follows: given three values, the combined distance ABC from *a* to *b* and *b* to *c* should be greater or equal to the distance AC between *a* and *c*. We tested whether the triangle inequality holds for the confusion distance by sampling 1 million randomly chosen triplets of languages. For all of these, ABC >= AC (to within 12 decimal places, which was the limit of the resolution of the data). This suggests that the triangle inequality holds in this data.

### Independent variables predicting confusion and accuracy

We tested a number of different factors that might predict whether two languages are confused for each other (being geographically close, their genealogy, similar phoneme inventories and similar lexicon). We also tested factors in relation to the rate at which the language was identified accurately (high quality audio clips, large global reach etc.).

In this section the different sources of information for these factors are described. All this information for the languages, clips and countries are available in the supporting information.

#### Factors predicting confusion

For *geographical closeness*, each language was identified with a point location (latitude and longitude according to the World Atlas of Language Structures [34], which intend to represent the cultural and/or historical center of the language). The log geographic distance between each language was calculated and converted to a distance matrix (this was highly correlated with geographic distances calculated from other sources, e.g. Glottolog).

Information on *language genealogy* was taken from Glottolog [12]. Genealogical distance between languages was calculated as the inverse of the number of nodes in common in the top four nodes in the Glottolog hierarchy.

*Similarity of phoneme inventories* was calculated based on inventories of segments from the Phonetics Information Base and Lexicon—database (PHOIBLE, [29]). This database contains phoneme inventories of the world’s languages, and 31 of the languages of the Great Language Game were represented and possible to compare. For this measure we only considered similarity of phoneme inventories. We did intend to include phonotactic features such as the World Atlas of Language Structures chapters 12, 14, 15 and 16 which deal with stress and syllable structure. However, information on these features are not avaible on very many of the GLG languages, so we had to discard those factors. Phonological difference between two languages was calculated as the proportion of common segments between them (see below for full method).

*Lexical similarity* was calculated using the Automated Similarity Judgement Program database (ASJP) [35]. We calculated the average, normalised distance between wordlists from a given pair of languages (the Levenshtein Distance Normalized & Divided, LDND, [36], see supporting information S6 Data for a list of word lists used). These wordlist are specifically constructed for inference about linguistic history, i.e., words that tend to be unstable in the transmission of languages through generations are excluded.

#### Factors predicting accuracy

We measured *audio quality of the speech samples in the game* using the Acoustic Diversity Index (ADI, [37]). This measure divides the audio file into 1000Hz bins, calculates the proportion of the signal in each bin which is above a given volume (-50 dBFS), then takes the Shannon index of these proportions. This gives a measure of the range of frequencies in the signal. Subjectively, low diversity samples sound unclear, poor-quality and noisy, like a bad telephone line (which is sometimes the actual context of recording). The mean ADI for recordings of each language was calculated (see supporting information S5 Data).

*Size of native (L1) and non-native (L2) speaker populations* for each language were taken from the Ethnologue [38] and [39]. The *proportion of L2 speakers* is simply L2/(L1 + L2).

The *linguistic diversity of the main country* where the language is spoken was measured using the Greenberg Diversity Index (GDI) from the Ethnologue [38]. This reflects the probability of two people from the same country speaking the same first language. The classification of what is the ‘main country’ of a language was taken from the Ethnologue, and is the same as was used for the measure of economic power.

*Number of countries language is spoken in* was taken from the Ethnologue [38].

We also coded for *language name transparency*—whether it is easy to match the language name with a country (usually the main country). For example, ‘Spanish’ can easily be associated with ‘Spain’, but it is harder to match ‘Urdu’ to India or Pakistan. Languages were classified (by HS and SR) as having a name with a transparent link to the main country or not. Alternative names for languages which do not appear to players were not considered.

*Economic power* can be measured by the Gross Domestic Product of the main country. We used the purchasing-power-parity (PPP) of the Gross Domestic Product (GDP) measured in billions of US dollars in 2012. This data has been compiled by Mikael Parkvall of Stockholm University, who kindly shared it with us.

We counted the *frequency of occurrence of the language name in Google Books* in English texts, and the Mandarin name of the langauge in Chinese texts (where “Chinese” here refers to Google’s definition, which in this case is the orthographic form and therefore not only Mandarin).

### Visualising confusion between languages

We use Neighbor-Nets to visualise the confusion between languages. Neighbor-Nets visualise distance matrices by iteratively joining nodes together to form a network. They have been used to explore the history of languages using synchronic data. For example, [40] compute a distance matrix between languages based on cognate words. The resulting Neighbor-Net reflected well-known historical splits in language families, but also clusters of areal phenomena resulting from languages of different genealogy influencing each other through contact (cf. [41]). However, they are best explained with reference to examples, so we explain further in the results section. We used the software package SplitsTree4 to create the Neighbor-Nets [42].

We also generated a binary tree from the confusion matrix using a hierarchical clustering algorithm (Ward’s algorithm, [43]).

### Identifying the most important factors predicting accuracy

**Fig 3:**
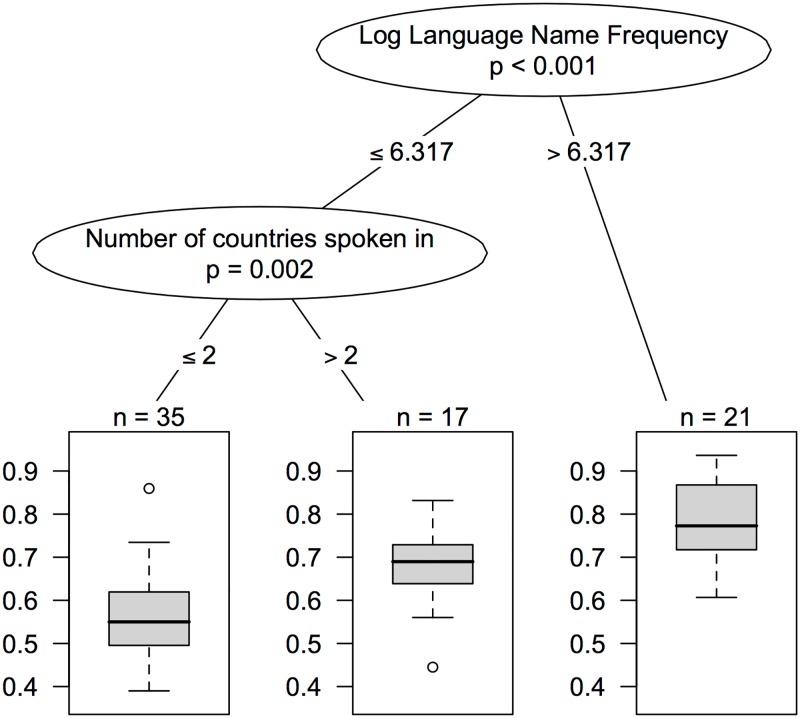
A binary decision tree showing the proportion of correct responses for languages in three categories.

Highly correlated independent variables can be problematic for regression analyses. The extent of the correlation can be assessed using the maximum variance inflation factor (VIF, the extent to which the variance of coefficients increase when adding variables into an ordinary least squares regression. If variables are correlated, then adding them in the same regression will increase the estimate variances and give a higher VIF, see [44]). For this analysis VIF = 6.6, which is considered an indication of highly colinear variables. Therefore, in order to assess the relative importance of different variables for contributing to the recognisability of a language, we conducted a random forest analysis which is robust to multicolinearity. Random forests (see [45]) is an approach to regression (and classification) based on decision trees. A decision tree is a hierarchy of yes/no-questions that optimally splits the data into subsets so that members inside a subset resemble each other. It is easiest to explain with an example. Fig 3 shows a decision tree predicting the accuracy of guesses from a number of predictor variables. The top node in the tree splits the GLG data into languages which are mentioned frequently and those which are mentioned infrequently (in Google Books). It then splits the infrequently mentioned languages into those which are spoken in many countries and those which are spoken in few countries. This tree divides the data into three sub-sets: frequently mentioned languages, infrequently mentioned languages spoken in many countries and infrequently mentioned languages spoken in few countries. The proportion of correct guesses decreases in each category. Variables used in questions nearer the top of the tree are have greater predictive power. A regression is then performed on each sub-set, which fits the data to some degree. The tree in Fig 3 accounts for about 50% of the variation in accuracy.

However, this is just a single possible tree. A ‘forest’ consisting of a number of randomly generated trees can be produced and evaluated. The importance of a variable can then be estimated as the aggregated ranking of the position of the variable in all trees. It is thus similar to stepwise regression in the sense that it considers a large number of models with different selections of variables. For our purposes, random forests provide a way of assessing the relative importance of variables when they are highly correlated. We implemented our analysis with the R package *party* [46].

### Identifying salient phonological cues

We also used conditional inference trees to investigate the effect of specifically linguistic factors on the accuracy of guesses. We transformed the PHOIBLE database [29] to represent the presence or absence of phonological segments. For each phonological segment, languages were classed as ‘same’ if they both had the same value (both languages have the segment or neither language has the segment), or ‘different’ if not. A conditional inference tree was then constructed that best splits the data according to these similarities and differences in order to model the confusion probabilities. That is, we estimate the most efficient series of yes/no-questions to ask about a pair of languages if one is trying to guess their probability of confusion. This tree could also be understood as an estimate of the sequence of questions (about phonological features) that players ask themselves when making a decision, and of the relative importance of the various features.

## Results

The final data includes more than 15 million guesses (after taking out guesses made by players with IP-addresses in a country where the target was official or de facto-official). The overall probability of guessing a language correctly was 70%. The language pair most likely to be confused is Punjabi and Kannada (Kannada is mistaken for Punjabi in 55% of trials where Punjabi is an option), and the language pair least likely to be confused is French and Vietnamese (Vietnamese is mistaken for French in 0.9% of trials where French is an option). French is the language that was identified correctly most often. The supporting information S2 Data includes a full list of confusion rates.

**Fig 4:**
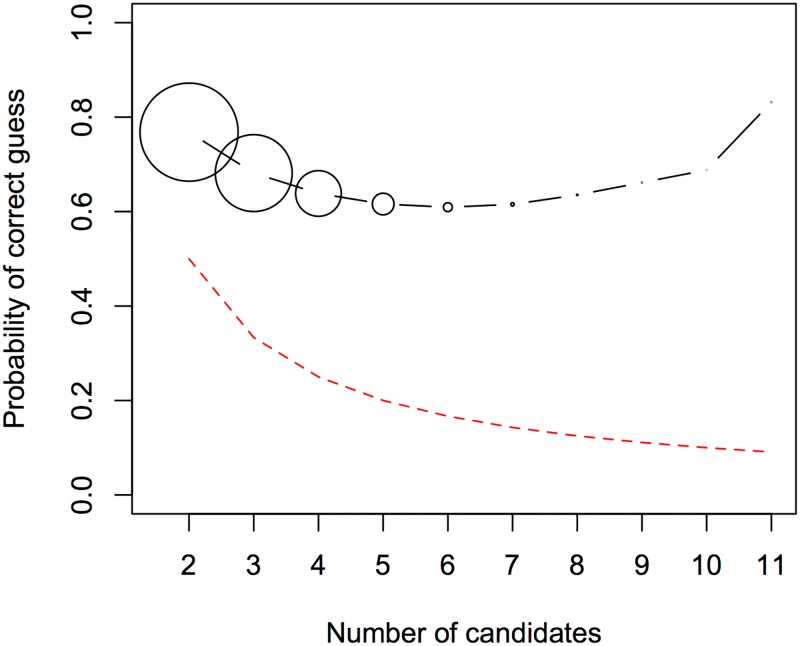
Proportion of correct responses by number of candidates. The proportion of trials guessed correctly as a function of the number of alternative choices. The size of the circles indicates the relative number of trials that had a given number of candidates. The red line indicates the proportion of correct responses expected by chance.

Fig 4 shows the probability of guessing the target language correctly given the number of alternative candidates, and demonstrates that players are performing above chance. Performance decreases until there are 6 alternatives, but then increases. Players presented with 11 candidate answers actually guess correctly more often than players guessing from 2 candidates. This is probably due to the structure of the game: context size keeps increasing, until the player has made 3 incorrect guesses, at which point the game stops. Therefore, only expert players experience a high number of alternatives. The impact of expert players on the data as a whole is small: only 2% of trials involve more than 6 alternatives.

In the following sections, we present the results of our investigations in relation to our research questions. The first questions relate to confusion while the latter relate to accuracy. The section ends with a summary of the results.

### Q1: Which languages are confused for which?

**Fig 5:**
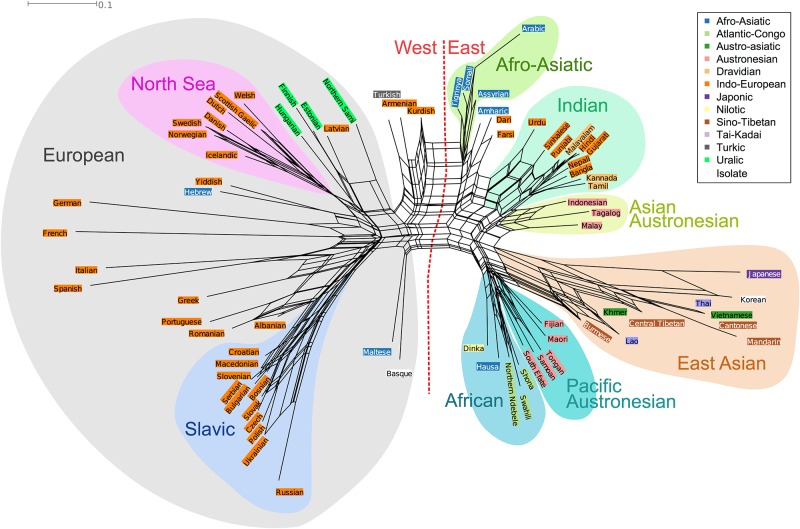
Neighbor-Net of confusion of the languages in the GLG.

In order to visualise the results, a Neighbor-Net was generated based on the confusion matrix for all guesses. Fig 5 shows the Neighbor-Net graph of the distance matrix from the GLG. The graph has been annotated for additional information; the colored highlighting of language names corresponds to language families and the polygons to geographical areas. First, we will introduce the concept of Neighbor-Net.

Each language is represented as a tip on a graph (next to a label with the language’s name). Tips are connected by a series of lines. The network is drawn so that for any two languages the length of the shortest path that connects them is approximately the distance between them in the input matrix. Therefore, two nodes that are connected by a short path are confused for each other more than two nodes that are connected by a long path. For example, there is a short distance between Tongan and Samoan, but a long distance between Tongan and Welsh. This means that Tongan is confused for Samoan more often than it is confused for Welsh. Languages like French and German are connected to the rest of the network by long lines, indicating that they have a high distance to any language, meaning that they are often guessed correctly. It is important to note that it is distance along the lines, not along the perimeter of the entire cluster that is key to reading a Neighbor-Net (i.e. the distance between the positions of two tips without travelling along lines is not meaningful).

One advantage of a Neighbor-Net is that it can display more complex relations between nodes, for instance a language being confused for two different groups of languages. The Neighbor-Net represents ‘confusability’ with a web of lines. For example, Armenian (Indo-European) and Turkish (Altaic) are often confused and have parallel lines connecting them making the total number of nodes that they share fewer than for example those shared by Turkish and Northern Sami (Uralic).

To appreciate the results, it is helpful to consider the extreme shapes that the graph could take. If every player guessed languages correctly almost all the time, then there would be little confusion and the Neighbor-Net would look like a star, with each language connected by an equally long line to a single central hub (similar to how German and French now stand out). If players guessed randomly, then the Neighbor-Net would look more like a web, with large areas of confusion and no clear meaningful clusters. In Fig 5, we see what would appear to be a moderate amount of confusion (delta score = 0.3, q-residual = 0.03), suggesting that players are not perfect at guessing languages, but not completely random in their confusion, since there are also clusters. For example, if players were reasonably good at guessing specific languages, but better at identifying Slavic languages in general from non-Slavic languages, then the Slavic languages would form their own ‘branch’ or cluster in the tree. In fact, that is exactly what we find! If the players were all historical linguists, making their judgments based on linguistic history (e.g. always guessing within the correct language family), then the clusters in the Neighbor-Net would reflect language families. We see some evidence for this, but also some counter-evidence. Similarly, if players were making judgments based only on phonology, then the Neighbor-Net would look like a Neighbor-Net produced with phonological distances, and if players were making judgments based on geographical location (e.g. they confuse languages that come from the same part of the world), then the Neighbor-Net would reflect geographical distances and, ideally, look similar to a geographical map of the world.

**Fig 6:**
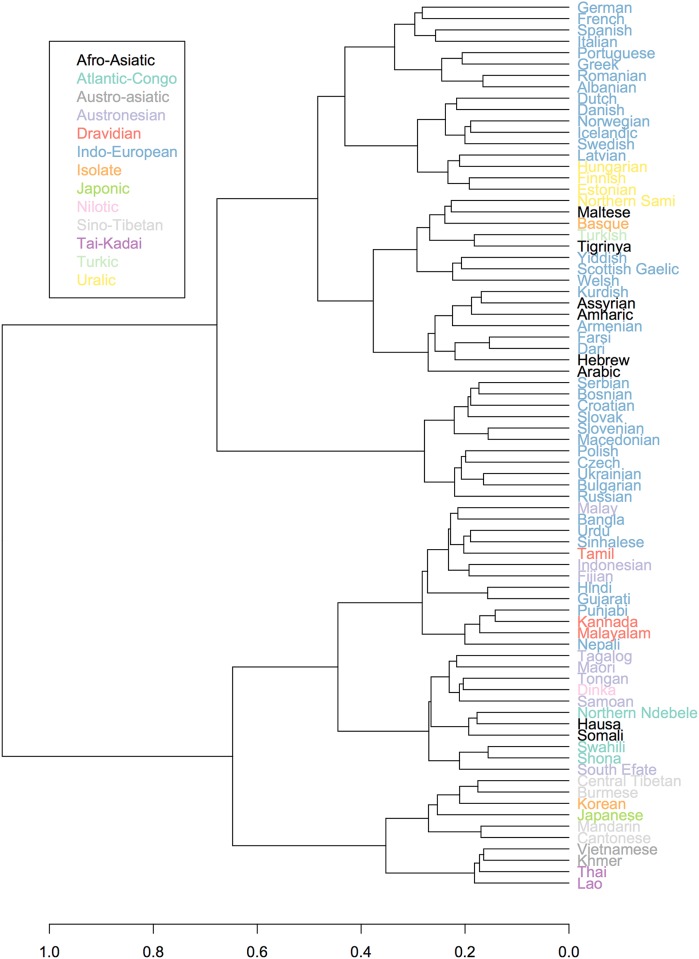
Binary tree based on the confusion data from GLG, excluding guesses from countries where the target language is official or *de facto*-official.

We can contrast visualisation of the Neighbor-Net with a binary branching tree. Fig 6 shows a tree calculated from the confusion distance matrix. These two visualizations complement each other by showing different relationships being displayed due to different properties of the graphs. Trees are undirected rooted graphs, showing exactly one relationship per node whereas a Neighbor-Net is a directed un-rooted graphs able to show multiple relationships. For example, the Neighbor-Net places Northern Sami, Latvian and Estonian relatively close (indicating conflicting signal), while the tree places Latvian and Estonian together, but Northern Sami in a more distant branch.

In the rest of this section on confusion, we explicitly test whether the distance matrix of player judgments correlates with genealogical, phonological or geographical distance, but from this very first visualization we can already make a few interesting observations.

The first major split that we see in the binary tree (Fig 6)-and also, less clearly, in the Neighbor-Net (Fig 5)-is between mostly languages of Europe on one side and languages mainly from Asia and Oceania on the other. The few languages of Africa present in the game (Amharic, Hausa, Arabic, Shona, Swahili, Northern Ndebele, Tigrinya and Dinka) are found on both sides. The languages of the Atlantic-Congo family (Shona, Swahili, Northern Ndebele) appear on the “Asia-Oceanic” side, but the Afro-Asiatic languages and in particular the Semitic ones are more complicated. The Afro-Asiatic and Semitic languages of Africa (Tigrinya, Arabic, Assyrian, Amharic) are found on the “European” side, together with Semitic Maltese and Hebrew (note that we are using the language names as they appear as an option in the game, i.e. “Hebrew” and we are referring to what is otherwise labeled as “Modern Hebrew”, not Ancient/Classical Hebrew). The other two Afro-Asiatic languages, Hausa (Chadic) and Somali (Cushitic), are found on the “Asia-Oceanic” side, close together with the Atlantic-Congo languages.

The east-west split can also be interpreted as a result of the bias in pool of players and the global fame of the languages, or more accurately lack thereof, considering that lesser known languages of Africa, Oceania and Asia are being lumped together.

The first split on the “European side” is between Slavic languages and the rest. The Indo-European languages, in particular French, German and Spanish, are often guessed correctly, there is less confusion there (see the supporting information S3 Data).

The Slavic languages form a coherent cluster, but the Romance languages do not in the same way. This is clearly illustrated both in the Neighbor-Net and the binary tree visualisations. There are only five Romance languages in our sample (Portuguese, Romanian, French, Spanish and Italian). French is most often guessed correctly overall (94%) and is therefore not particularly close to any of the other languages in the sample (the same applies to German). Spanish and Italian are also very often guessed correctly; if anything they are confused with each other. However, Portuguese and Romanian are associated with Greek and Albanian and also Slavic languages. In the case of Romanian this is most likely due to similarities in phonology with Slavic due to extensive contact (e.g. [47], p. 11–12), but perhaps also players’ cultural knowledge of Eastern Europe as a region of much cultural contact, both historically and in recent times (Soviet Union).

In the case of Portuguese, however, the similarities with the Slavic languages cannot be due to contact or culture as with Romanian, but rather coincidental phonological similarity. Portuguese shares 58% of its segments with Croatian and 53% of its segments with Polish (data from PHOIBLE). This is within the top 1% of most similar segment inventories in our data (mean shared segments = 26%). Besides the fact that Portuguese and Romanian both appear close to the Slavic languages, as do Albanian, they also are confused for each other. This is not surprising to the authors of this paper. One possible explanation is that Romanian and Portuguese are two Romance languages that are often perceived as “sounding Slavic”, due, at least, in part to the aforementioned contact (Romanian-Slavic) and the similarities in phonology (Portuguese-Slavic).

The Indo-European languages of Europe are more often associated with the non-related languages of the Uralic or Afro-Asiatic families than their Indo-European cousins in South Asia: Hindi, Bangla, Sinhalese, Gujarati and Punjabi. In fact, we also find Turkish and Basque closer to the Indo-European languages of Europe. Basque is an isolate language spoken in Spain and France, it has no known relatives. It is unclear if it is similarity due to contact that triggered this confusion or cultural knowledge of where it is spoken. In our comparison of phoneme inventories Turkish and Basque are similar to German and Spanish, respectively.

The Indo-European languages of the Indic and Iranian genera are usually clustered with other languages in their geographical vicinity, i.e. in Southern and Western Asia. Farsi and Dari stand out among the Indo-European languages spoken in Asia as being slightly closer to the western Indo-European languages and to Semitic languages than to other eastern Indo-European languages. This can also be seen in the binary tree (Fig 6) where Farsi and Dari are more tightly clustered with Hebrew and Armenian than with languages of South Asia.

The four Uralic languages appear close in both visualisations, with the interesting addition of Latvian. Latvian is an Indo-European language of the Baltic branch, spoken in close contact to Estonian (Uralic). This pattern might be due to contact influence in the phonology of Latvian. It is also important to take into account that there are no other Baltic languages in the sample that Latvian could be associated with, i.e. Lithuanian for example is not included. Based on genealogy one might expect Latvian as a Baltic language to appear closer to the Slavic languages [48] (page 222) [49]. However, this connection is more than 3,000 years old and the shared features are most likely not salient to players in these short speech samples. The cultural knowledge that Latvian is spoken close to Uralic languages and/or contact influence from these languages is probably a stronger factor.

On the whole, there is a lot of conflicting signal in the Slavic cluster, indicating that players confuse Slavic languages for each another. However, this is not symmetrical: every Slavic language is more often confused with Russian than the other way around (this is discussed in more detail later in our paper), resulting in Russian being more removed from the other Slavic languages in the Neighbor-Net visualisation. This indicates that Russian is the ‘prototypical’ Slavic, or at least the one that players associate most with Slavic sounds.

It is important to note that Slavic is the most well-represented subfamily/genus in the entire language sample; there are 11 Slavic languages, compared to 7 Germanic and 5 Romance (take into account that, as mentioned earlier, Bosnian, Serbian, and Croatian are classified as 3 different languages in Ethnologue, Glottolog and in the Great Language Game, but that this is not necessarily done everywhere else). For more information on the representation of families and genera, see supporting information S1 Data.

Another interesting observation is the clustering of the Austronesian languages. In both Figs 6 and 5, they are split between Austronesian languages of Oceania (Maori, Tongan, Samoan, Fijian and South Efate) and Western Austronesian languages (Malay, Indonesian and Tagalog). The Oceanic languages are associated with some of the languages of Africa in the sample (Dinka of the Nilo-Saharan family, Northern Ndebele, Shona and Swahili of the Atlantic-Congo family and one member of the Afro-Asiatic family: Hausa). The Western Austronesian languages are found to be closer to languages of East Asia and India, particularly Dravidian languages.

In contrast to a Neighbor-Net, a binary tree forces a language to appear with one particular group, which can lead to strange clusters. In the binary tree the Fijian (Oceanic, Austronesian) appears alongside Malayic Austronesian languages and languages of the Indian continent. This is likely to be an artefact of the binary tree being unable to handle the complex relationships in the data; in the Neighbor-Net, Fijian appears with the other Oceanic Austronesian languages, which intuitively makes more sense.

Now we turn to Korean and Japanese. The possibility of a genealogical connection between Korean and Japanese has long been a topic of debate in linguistics [50]. It is widely accepted that Japanese forms a small family together with the languages of the Ryukyu islands (these are however not present in the game)—but is Korean to be included there, viewed as an isolate language, or related to another grouping? There are researchers who group Korean and Japanese as part of an even greater family—Altaic. The macro-Altaic hypothesis (we use the term ‘macro-Altaic’ to refer to the Altaic hypothesis that includes Tungusic, Mongolic, Turkic, Japanese, Ainu and Korean, as opposed to simply ‘Altaic’ or ‘micro-Altaic’ that only covers Tungusic, Mongolic and Turkic) also includes Mongolian and Turkish [51]. Glottolog, which was used as the information source for linguistic genealogy in this paper, does not place Korean and Japanese in the same family, nor does the Ethnologue. It is unclear if the confusion in the GLG between Korean and Japanese is due to phonological similarity, geographical proximity, genealogy or players’ cultural knowledge. Either way, the players of the game have made a connection between Japanese and Korean. In fact, they confuse several languages of East Asia, South Asia and Mainland Southeast Asia with each other, despite these languages being of different language families. Players are however not confusing Japanese and Korean with the only other macro-Altaic member present in the game—Turkish—particularly often.

This cluster of languages from East Asia (Cantonese, Mandarin, Japanese and Korean), South Asia (Central Tibetan) and Mainland Southeast Asia (Khmer, Burmese, Lao and Thai) is quite clearly visualised in the Neighbor-Net. This cluster is made up of languages from at least 4 different families: Sino-Tibetan, Austro-Asiatic, Tai-Kadai, Japonic and the isolate Korean. Several of these languages are in a well-known contact area and share many features, in particular the salient features of having tone and sesquisyllabicity [52, 53]. Khmer and Korean are the only languages in the cluster that lack tone (there are recent studies showing that varieties of Khmer and Korean are developing tone [54, 55], but it is unclear how salient these distinctions are to a non-native speaker as it is a change in progress with restricted distribution). Besides sharing similar features, they might also be lumped together because players (in particular those from Europe, the US and Australia) may perceive these languages and people as a cultural unit (“Asian”).

Languages of Africa are less coherent. The Atlantic-Congo languages cluster together, as do some of the Afro-Asiatic languages, but the two groups are far apart from each other.

At a macro-scale, the clustering appears to be partly based on geography. For example, the graph as a whole splits into languages from the ‘East’ and ‘West’, with coherent clusters for languages for Europe, India and South-East Asia. Within the European languages there appears to be a sub-group composed of languages bordering the North Sea (Swedish, Norwegian, Danish, Icelandic, Dutch, Scottish Gaelic and Welsh). Since there are both Germanic and Celtic languages in this group, geography seems a more parsimonious explanation than genealogy. One possible explanation for the cluster is similarity of phonologies due to contact along sea trading routes (e.g. [56]).

There are some patterns that are inconsistent with language family history. Yiddish for example (an Indo-European language) shares a branch with Hebrew (an Afro-Asiatic language). Yiddish and Hebrew may be of different language families, but they are both languages spoken by a mainly Jewish population and this may form part of players’ cultural knowledge of these languages. In addition, during the revival of Hebrew, Yiddish had a considerable influence on the phonology [57, 58].

If players are using cultural knowledge such as shared culture, history and religion of speakers of Yiddish and Hebrew, then we might expect Neighbor-Nets constructed from judgments by players from different parts of the world to look different. We constructed Neighbor-Nets for several geographic regions (continents, according to the ISO convention) and we do find some differences: players from North America associated Yiddish and Hebrew more with each other than players from Africa or Asia do. In fact, African players confused Hebrew and other Afro-Asiatic languages more often than Hebrew and German—which is more in line with language history. Neighbor-Nets constructed from responses from each separate continent can be found in supporting information S4 Data.

We also calculated a confusion matrix for responses from each country with more than 50,000 data points (42 countries). All pairs of countries were significantly correlated in their confusions (Mantel p < 0.01, adjusted for multiple comparisons using Holm’s method [59]), but the correlation strengths vary from r = 0.25 to r = 0.95. This suggests that the judgements of players from different countries are more similar that one would expect by chance, but there are still subtle differences. The country pairs with the highest similarity are the UK with the USA, and Argentina with Mexico. The country pairs with the lowest similarity are France with China, and Poland with Slovenia. From these examples, one might make two predictions about what would predict similarity in judgements: the amount of cultural similarity and geographic proximity. However, preliminary post-hoc analyses found no statistical support for this. Confusion distance actually has a weak negative correlation with geographic distance between country capitals (Mantel r = −0.13, p = 0.09). Also, the proportional migration between two countries does not predict the similarity of judgements between them (data from [60], Mantel r = −0.08, p = 0.24). Supporting information S4 Data includes a Neighbor-Net produced from the judgement differences between countries.

#### Phonological distance between the languages

**Fig 7:**
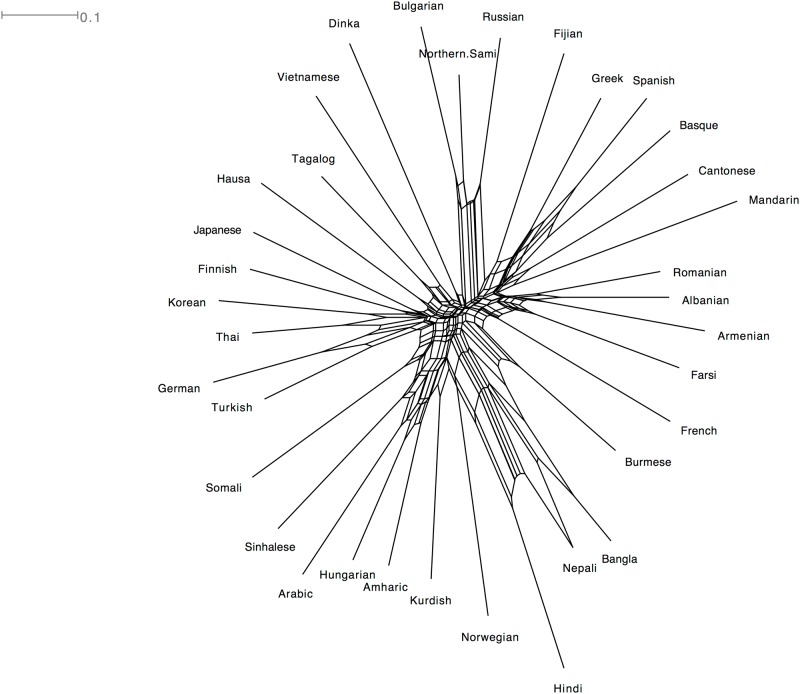
Neighbor-Net of the differences in phoneme inventories of 31 of the GLG-languages. Distances are defined as the inverse of the proportion of phonemes shared between two languages.

Does the Neighbor-Net of confusion simply reflect the differences in phonologies between languages? Fig 7 shows a Neighbor-Net constructed from phoneme inventory distances. It has some similarities to the players’ confusion in the game, for example, it clusters Romanian an Albanian and these two languages do appear close both in the Neighbor-Net and the binary tree of confusion. It differs to a larger extent in other instances though, for example by associating Hungarian with Amharic instead of the more probable partner of Finnish. Overall, it would seem that it does not represent a meaningful clustering of the languages. The importance of this factor is explored in further details in the following section.

### Q2: Are there any asymmetries of confusion?

The confusion rates were relatively symmetric: the confusability of opposing pairs correlated at r = 0.61. The pair of languages with the biggest asymmetry is Tigrinya and Arabic: Tigrinya is 6.6 times more likely to be confused for Arabic than the other way around. Danish, Hungarian and Turkish are between 5 and 6 times more likely to be confused for Vietnamese than the other way around. Ukrainian, Slovak and Czech are also amongst the languages with the largest asymmetry, in this case with Russian, as explained earlier.

On the other hand, several language pairs show high symmetry. The following pairs were equally likely to be confused for each other: Welsh and Samoan; Burmese and Romanian; Albanian and Burmese; Kurdish and Yiddish.

Perhaps languages that display an asymmetry of mutual intelligibility also show an asymmetry of confusion by players in this game? However, certain well-known mutual intelligibility asymmetries by native speakers, such as Swedish and Danish [61], are not reflected in the confusion asymmetry in our data, which is based on judgments from players in countries where these languages are not official or *de facto*-official. Swedish is mistaken for Danish only 1.1 times more often than Danish is mistaken for Swedish. Danish and Norwegian are similarly symmetric.

Table 2 shows that a given Slavic language is more likely to be confused for Russian, than Russian is to be confused for it. This supports the idea suggested above that Russian is seen as the ‘prototypical’, or at least the best-known, Slavic language. It might also reflect mutual intelligibility asymmetries, i.e. a scenario where speakers of language X understands speakers of language Y better than the other way around (c.f. [61]). Unfortunately, we have no studies of mutual intelligibility of Slavic languages for comparison. It may be that asymmetries of confusion and mutual intelligibility are not correlated with each other, but rather both dependent on another variable (population size, fame etc). However, this cannot be known in this study.

**Table 2:** Asymmetries in the guessing of Slavic languages.

Language	confused for Russian	Russian confused for language	Asymmetry
Ukrainian	40.5%	25.4%	1.59
Bosnian	23.8%	14.5%	1.64
Croatian	20.0%	12.1%	1.65
Polish	31.7%	17.3%	1.83
Serbian	31.3%	15.7%	1.99
Slovene	27.4%	13.6%	2.02
Czech	32.2%	15.6%	2.06
Slovak	31.7%	15.3%	2.07
Bulgarian	37.9%	16.3%	2.33
Macedonian	35.5%	10.2%	3.49

### Q3: What factors can predict player confusions?

Based on the visualisations presented above (Figs 5, 6 and 7), player judgements appear to be partly compatible with the historical relationships between languages, partly with how similar they sound, partly with geographical relations, and partly with cultural knowledge. It is difficult to tease these effects apart only using the visualisations, so below we perform explicit tests of the different factors proposed in our research questions.

As described above, we produced distance matrices for language confusion, geographic distance, genealogical distance, phonological distance and lexical distance (data available in supporting information S7 Data).

Tables 3 and 4 show the results of the Mantel tests comparing the distance matrices (calculated using the R package *ecodist*, though there were no differences when computing the correlations with implementations in other R packages). Geographic distance is significantly correlated with genealogical distance, lexical distance and phonological distance (languages further apart geographically tend to be more different). Lexical distance is also correlated with genealogical distance, which is to be expected given that the ASJP database was specifically aimed at inferring language history.

**Table 3:** Mantel correlations between independent variables, with probabilities in parentheses.

	Geographic	Genealogical	Lexical
Genealogical	0.49 (0.0001)		
Lexical	0.47 (0.0001)	0.67 (0.0001)	
Phonological	0.15 (0.021)	-0.08 (0.38)	-0.01 (0.86)

**Table 4:** Mantel correlations between independent variables and GLG confusion distance. Calculated using all language pairs.

Control For			Genealogical		Geography		Both	
	Mantel r	p	Mantel r	p	Mantel r	p	Mantel r	p
Geographic	-0.36	0.0001	-0.28	0.0001				
Genealogical	-0.14	0.012			0.01	0.88		
Phonological	-0.21	0.0095	-0.2	0.03	-0.17	0.074	-0.16	0.081
Lexical	-0.36	0.0001	-0.05	0.093	-0.03	0.49	-0.04	0.16

Languages were confused more often if they were closer in terms of historical relationship, geographical location, phonological overlap or in terms of their lexicon.

Because some of the independent variables are correlated, partial Mantel tests were used to assess the independence of the correlation of each variable with the GLG judgements while controlling for the other variables. The correlation with geography remained significant when controlling for the influence of genealogy, but not vice-versa. The correlation with phonological distance remained marginally significant even when controlling for both genealogy and geography (p = 0.08).

These results suggest that participants are better able to distinguish languages if they are far apart geographically or have very different phoneme inventories. While there are correlations between players’ judgements and language genealogy, these are mainly driven by covariation with geographic distance. It should be noted that the genealogical measure used above (distance in the Glottolog hierarchy) was quite coarse, and better measures may be available if more detailed trees of language history with branch lengths can be calculated (see [62]).

### Q4: What factors can predict players’ accuracy?

We explore various factors which affect accuracy, starting with a general overview of differences between countries.

#### Success according to players’ country

**Fig 8:**
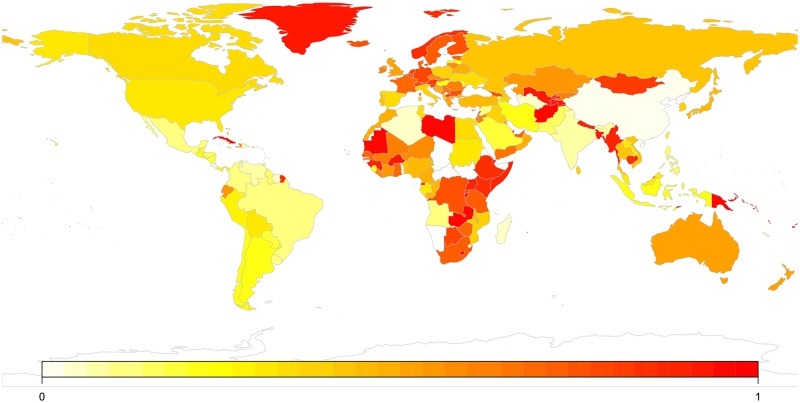
Proportion of correct responses by geographic region. The map was created in R using the package *maps* [26].

Table 5 shows the distribution of percentage of correct guesses over the top-10 and bottom-10 countries (excluding countries with less than 5000 data points). Fig 8 shows the distribution of correct guesses across the world. 8 of the top 10 countries are in Europe, while 7 of the bottom 10 countries are in Latin America. When considering this table, it is important to remember that most of the languages of the sample were European, most players were European or probably at least Anglophone, and that the language sample over-represent the linguistic diversity of Australia, Unites States and the United Kingdom.

**Table 5:** Rate of accurate guesses by country, top 10 and bottom 10 ranked by percentage of correct guesses.

Country	Percentage of correct guesses	Total number of guesses	Number of correct guesses
Luxembourg	75.0%	14,723	11,042
Slovenia	74.8%	60,172	45,009
Norway	74.7%	140,529	104,975
Kenya	74.6%	5,242	3,911
Austria	73.4%	131,755	96,708
Macedonia	73.3%	30,487	22,347
Lebanon	73.1%	6,414	4,689
Switzerland	73.1%	126,212	92,261
Netherlands	73.1%	324,261	237,035
Germany	73.1%	756,166	552,757
…			
Philippines	66.7%	22,831	15,228
Brazil	66.3%	208,601	138,302
Uruguay	66.2%	8,430	5580
Colombia	66.1%	25,736	17,011
Puerto Rico	66.1%	5,689	3,760
Mexico	65.8%	85,312	56,135
India	65.7%	69,560	45,701
Venezuela	65.7%	10,282	6,755
Costa Rica	65.4%	11,505	7,524
China	60.7%	56,452	34,266

#### Audio quality, global reach and language name transparency

In this section we test different factors influencing the accuracy of the guesses: acoustic quality of the speech samples, proportion of L2 speakers, size of total L1 speaker population, linguistic diversity of main country, number of countries the language is spoken in, language name transparency, economic power of main country, and frequency of occurrence of the language name in written materials.

The acoustic diversity of the speech samples varied from 0.82 to 2.20. There was a wide range in how accurately each recording was guessed correctly (31% to 97%). Samples with lower acoustic diversity were significantly less likely to be guessed correctly (r = 0.29, t = 6.1, df = 398, p < 0.001).

For the analysis of correct guesses, the mean acoustic diversity for each language was calculated (the mean and median for each language are highly correlated, r = 0.94). The language with the highest average acoustic diversity was Hebrew (mean = 2.12), and the lowest average acoustic diversity was Punjabi (mean = 1.10).

The sample with lowest diversity was: http://media.greatlanguagegame.com/samples/d8a4ee3f4b430af016b2dc5902fccd75.mp3 and the sample with highest diversity: http://media.greatlanguagegame.com/samples/3583f583d0f96068e16cb55d9c672d2d.mp3.

#### Q5: Are linguistic or non-linguistic factors more important in predicting accuracy?

The random forests analysis predicted the probability of a language being guessed correctly using the following variables: proportion of L2 speakers (L2/(L1+L2)), total L1 population size, linguistic diversity of main country, number of countries in which the language is spoken, language name transparency, economic power of main country and frequency of occurrence of language name in written materials (Google Books). Among these, L1 population size, economic power and frequency of language name were log transformed to avoid skewed distributions. There is data for all 78 languages on all of these variables, except for proportion of L2 speakers, where we only have data on 44 languages.

The models used 5 variables in each tree for 1000 trees. Conditional variable importance was calculated. The random forests model was used to predict the rates of accuracy, and the predictions were found to be highly correlated with the actual values (r = 0.81, suggesting around 66% of variance accounted for).

**Fig 9:**
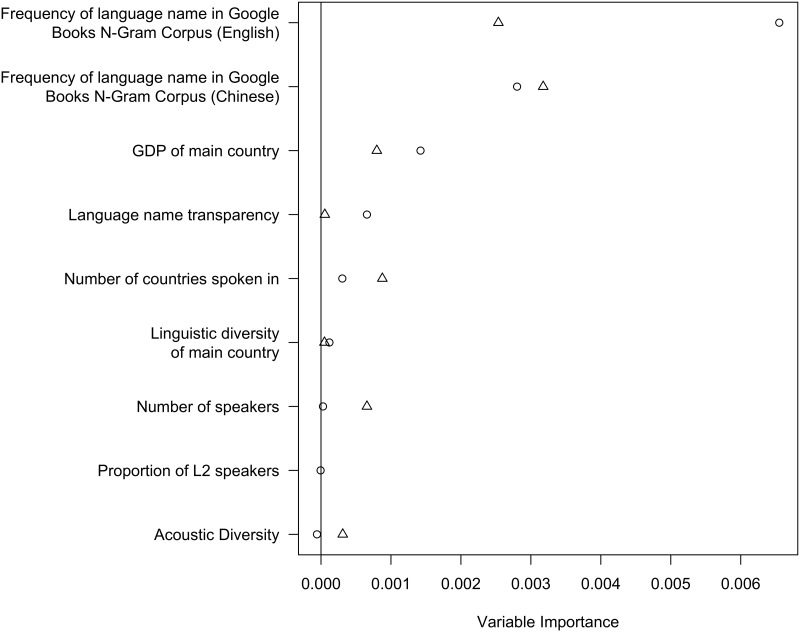
Variable importance for analysis 1 (including proportion of L2 speakers, circles) and analysis 2 (excluding proportion of L2 speakers, triangles).

**Fig 10:**
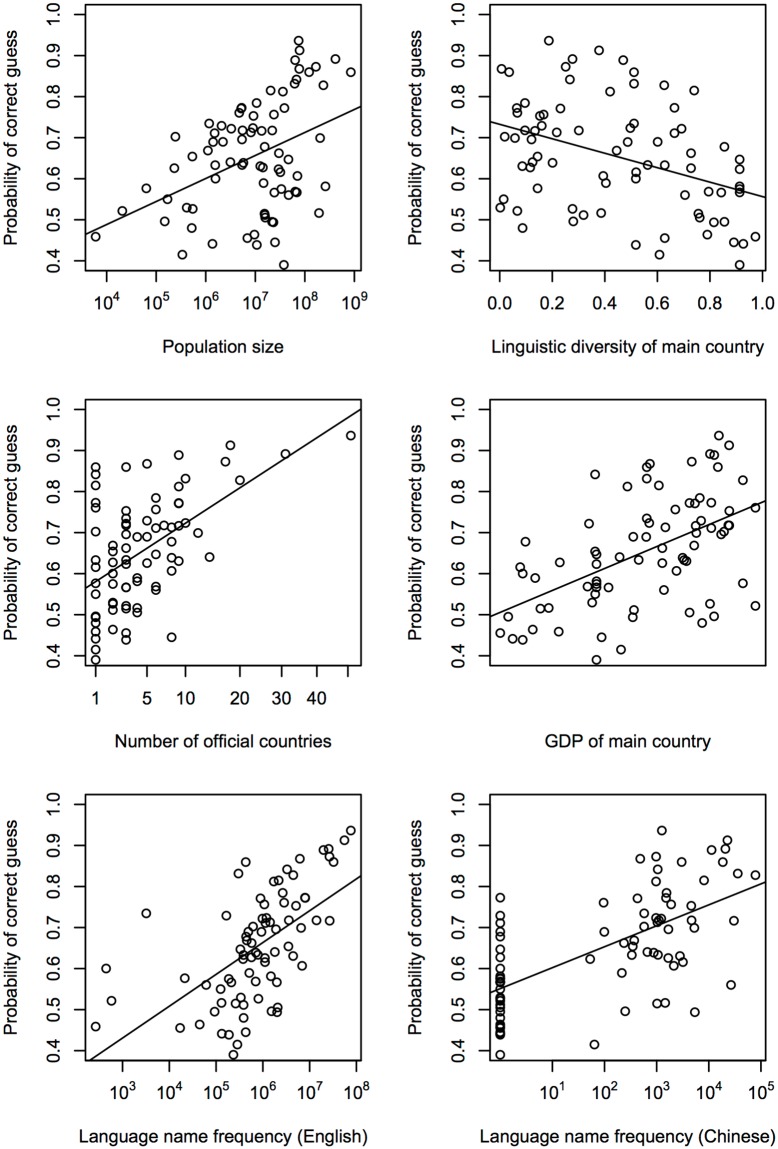
Graphs of correlations between six different variables and the accuracy of the guesses.

Table 6 and Fig 9 show the results (under ‘analysis 1’). The variable with the highest importance is the frequency of the language name in the English Google N-gram Corpus, followed by the name frequency in the Chinese Google N-gram Corpus. The next most influential variable is the economic power (GDP, PPP) of the main country of the language, followed by language name transparency and the number of countries the language is spoken in. The linguistic diversity of the main country and the number of L1 speakers are less important, with the proportion of L2 speakers and the acoustic diversity of samples being the least important variables. Some graphs of the relations between variables are shown in Fig 10.

**Table 6:** Correlation and variable importance for different variables predicting the probability of correctly guessing a language. Analysis 1 includes proportion of L2 speakers, while analysis 2 does not.

Variable	Correlation with correct guess (analysis 1)	Variable Importance (analysis 1) (x10000)	Variable Importance (analysis 2) (x10000)
Acoustic Diversity	0.27	-0.57	3.11
Proportion of L2 speakers (L1/(L1+L2))	-0.11	-0.06	(not included)
Number of speakers	0.32	0.29	6.57
Linguistic diversity of main country	-0.47	1.20	0.50
Number of countries spoken in	0.51	3.05	8.77
Language name transparency	0.50	6.57	0.54
economic power (GDP, PPP) of main country	0.56	14.23	7.98
Frequency of language name in Google Books N-Gram Corpus (Chinese)	0.58	28.03	31.76
Frequency of language name in Google Books N-Gram Corpus (English)	0.74	65.51	25.36

Since L2 speaker proportion is not available for some languages, the analysis was re-run excluding this variable, which allowed 73 languages to be analysed. The values for analysis 2 are shown in Table 6. In this analysis, the frequency of the language name in Chinese is ranked as most important. This is possibly due to the Chinese frequency measure being coarser than the English measure (some languages were not observed in the Chinese corpus), making it easier to divide the languages into groups using a binary tree. Also, the mean acoustic diversity of each language’s recordings and the number of official countries each is spoken in are ranked as relatively more important than they were in analysis 1.

Overall, languages are guessed correctly with a higher accuracy if they: have a higher frequency in the Google N-gram corpus; come from a country with low linguistic diversity and much economic power (GDP, PPP); are spoken by many people; are spoken in many countries as an official language; have a geographically transparent name; and have been represented using speech samples with high acoustic diversity.

These results show that many non-linguistic factors can predict the probability of guessing a language correctly, one of the most important ones being how often the language is talked about.

#### Q6: Are some phonological cues more important than others in predicting accuracy?

**Fig 11:**
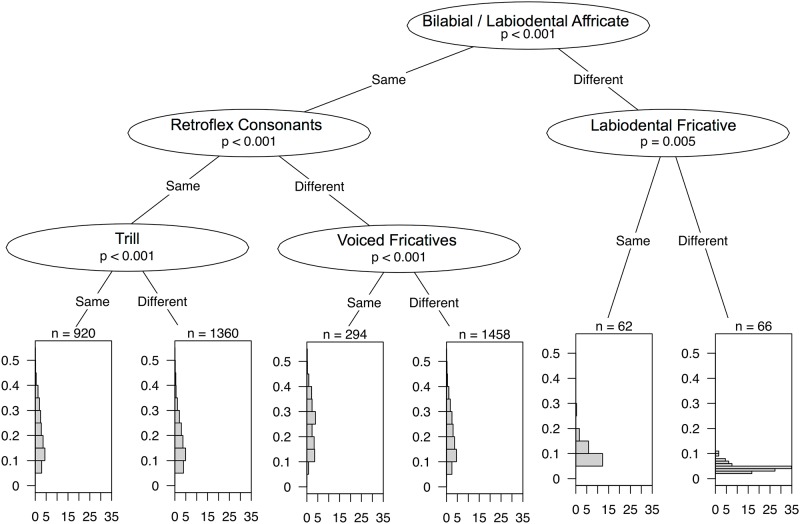
Binary classification tree for phonological differences between languages, with the probability of confusing two languages indicated at each leaf.

We produced a conditional inference tree predicting accuracy by presence or absence of phonological segments. An unrestricted algorithm returned a tree with 9 levels which accounted for around 18% of the variance in the confusion probabilities. This is a poor fit, or indicates there is a lot of noise in the data. However, some sensible results are obtained: Fig 11 shows the first three levels of this tree. Features lower down on the tree have less of an impact. Moving left in the decision tree means that the languages are more similar (harder to distinguish), while moving right means they are more different. The graphs at the leaves of the tree show the distribution of confusability. As one moves to the left, the probability of confusion increases.

The first divide is that of the presence/absence of the bilabial/labiodental affricate. Affricates are consonants that are initially produced as stops and have a fricative release. The only language in the sample with this phoneme is German. German is also the second most accurately identified language in the entire game (after French). This means that it is more likely that this distinction is less relevant and that it is being picked up by the tree because it occurs in a very recognizable language that might be easy to distinguish for other reasons as well.

The next divide is that of the presence/absence of retroflex consonants. These are consonants that are articulated by placing the tip of the tongue between the alveolar ridge and the hard palate. Retroflexes are an areal feature, found throughout most of South Asia. The importance of this feature may explain the finding that players were likely to confuse Indo-European languages from India with non-Indo-European languages of South Asia, rather than with other Indo-European languages.

The other distinctions attempt to single out uncommon phonological features. For example, trills ([r, R]) are not very common, making them a salient distinguishing feature. Labiodental fricatives ([f] and [v] and variations thereof) are quite common in the world’s languages, so perhaps the absence of these sounds is a salient cue for distinguishing between languages. However, it is unclear whether players would notice the absence of a segment in 20 seconds of audio (e.g. [f] or [v] occur in only 86% of 20-second samples from the Switchboard corpus of English conversation [63], total of 4524 samples).

### Summary of results

In this paper we have investigated the results of an online game where people have to identify a language from an audio clip of speech. We explored what factors may predict success in this task, and factors predicting confusion between the languages, including linguistic and non-linguistic factors.

Overall, players guessed correctly at a level well above chance—on average getting 70% of guesses correct. The factor investigated that most clearly correlated with accuracy was those relating to the ‘global fame’ of the language: how frequent the language name appears in Google Books, economic power etc. These reflect results of some previous experiments on more limited samples [18, 24].

There were smaller effects for how easy it was to connect a language name with a country (‘language name transparency’), the quality of the audio recording, the number of countries a language is spoken in, the number of speakers a language has and the number of L2 speakers.

We found that the probability of confusing one language for another was correlated with objective measures of genealogical, geographic, phonological and lexical distance. However, these effects were not all independent from one another. When controlling for geographic distance, the correlation with genealogy disappears and the correlation with both phonological and lexical difference weakens, suggesting that the main relationship is with geographic distance. This relates to some findings which show that linguistic distances vary continuously over geographic space [64, 65].

Our analysis of phonological similarity proved somewhat difficult, since we could only obtain data for a subset of languages. The Neighbor-Net constructed based on these similarities did not appear very similar to the confusion rates, except that Armenian, Albanian and Romanian appeared close. We tried to identify phonological cues that players may have been using. We found that confusion was less likely for languages which differed in the presence or absence of salient phonological segments such as bilabial affricates, labiodental fricatives and retroflex consonants.

We also found some evidence for differences between players from different countries, though the effects were weak.

## Discussion

In this study, we looked at the factors that influence perception of similarity in a global sample of languages and participants. We found some evidence that people in different countries make different judgements and that cultural knowledge and linguistic experience influence judgements of similarity and differences. However, confusion between languages is common, especially for languages that share a history of contact. This reflects the fact that the languages we observe in the world today are a product of cultural evolution: linguistic elements compete against each other to be ‘replicated’ in every utterance we produce [66], and the elements that survive are spread through time and space, mingling between groups of speakers and crossing language boundaries. Therefore, when we recognise similarities in the way people speak, we are often recognising shared history.

Different selection pressures may affect this evolutionary process, such as demands on processing or communicative needs of speakers. Perception is another clear selection pressure, but little is known about how humans recognise variation between languages that they are not familiar with. If perception is influenced by experience, and if the processes of language change are influenced by the biases in auditory perception, then the selection pressures may be different in different linguistic contexts. For example, in one case the prosody of a language might mark it as different from others, while in another context differences in lexicon might be more salient. Languages in competition with each other may change to emphasise the most salient differences between them. There is some evidence from artificial language learning experiments that linguistic diversity can be driven by the need to differentiate between cultural groups [5]. This might suggest that languages adapt to their local linguistic context, in addition to more general, uniform pressures such as memory limitations or basic pragmatic needs.

In this case, an important part of understanding language change and the emergence of diversity is understanding which similarities and differences are salient for speakers—both outsiders and in-group members. Understanding subjective differences in perception could offer an insight into what aspects of language or culture are salient in different contexts, and will therefore be oriented to and become a locus of change.

The data came from an online game which was designed to draw a large range of particpants and be fun to play. While this provides a large sample size, there are also weak points. Key improvements for a research perspective include eliciting more detailed data about the player’s linguistic background, limiting cultural knowledge by getting players to compare audio clips directly (rather than matching audio with a label) and making the game accessible to a wider range of speakers. Peter Withers, Seán Roberts and Hedvig Skirgård have developed a new version of this game called “LingQuest”, together with the Language In Interaction-consortium (https://www.languageininteraction.nl/lingquest.html, https://github.com/languageininteraction/LanguageMemoryApp). This new version presents players with multiple audio clips from a wider range of languages (including rare and endangered languages) and asks them to identify which is the pair of clips from the same language. It also gathers more information about the players and is available in more language besides English. We look forward to investigating the new data that emerges from this. More generally, we hope this study demonstrates how gamifying linguistic judgement tasks and massive online participation can be useful tools for exploring the similarities and differences between languages.

## Supporting information

S1 Appendix Note on stimulus selection. (PDF)

S1 Data Language data. This includes data on population sizes, country-level data, language name frequency, mean acoustic diversity, and language codes.
(ZIP)

S2 Data Confusion probabilities. The probability of confusion each language for every other language. Row names are identical to column names. To find the probability of confusing language A for language B, look up row A, column B.
(ZIP)

S3 Data Guessing accuracy for each language. This includes which languages are most often and least often confused for each language.
(ZIP)

S4 Data Neighbor-Nets for each continent. (PDF)

S5 Data Audio diversity and quality measures for each speech sample. (ZIP)

S6 Data A list of which ASJP doculects were used to represent the lexicon of the languages in the analyses. (ZIP)

S7 Data Distance matrices and an R script for calculating the correlation between different measures (Mantel tests). (ZIP)

S8 Data Raw results from the Great Language Game. This large file is available through FigShare: http://dx.doi.org/10.6084/m9.figshare.3512090.
(ZIP)
