# Supplementary material for: Why are some languages confused for others? Investigating data from the Great Language Game
Source: PLoS One. 2017 Apr 5;12(4):e0165934. doi: 10.1371/journal.pone.0165934 (PMC5381764; doi:10.1371/journal.pone.0165934)
Supplement: S1 Appendix — (PDF) [file pone.0165934.s001.pdf]

S1 Appendix: Note on stimulus selection

The audio clips are chosen unpredictably, but not uniformly. Languages are sorted by the accuracy of the global player pool to date, and then a linear likelihood ramp is applied based on the player's current difficulty level. This means that well-recognised languages such as French are more likely to appear earlier, and more poorly-recognised languages such as Shona are more likely to appear once players have already identified many languages correctly. This introduces complications into the results, meaning that players are more often exposed to languages which are more often guessed correctly. The language that appeared as the target most often appeared around 4 times more often than the language that appeared least often (325,915 times compared to 78,858 times). However, controlling for this is difficult since the weighting changed dynamically throughout the game's history. The weighting means that `harder' language are more likely to appear as targets for `expert' players, players that are less likely to guess wrong and randomly. In this case, the potentially worse estimates from less exposure may be offset by ratings from better performing players (we note that there is a tension between design decisions that make an `attractive game' to players and those that make a `good scientific experiment'). This is the game's other difficulty mechanic (the first being the increasing number of distractors) and perhaps also serves as a way of not scaring away players from the start. Despite these problems, the assumption that target languages are randomly chosen is reasonable, since, on average, a player will have little experience with most languages in the game.
