# Supplementary material for: Why are some languages confused for others? Investigating data from the Great Language Game
Source: PLoS One. 2017 Apr 5;12(4):e0165934. doi: 10.1371/journal.pone.0165934 (PMC5381764; doi:10.1371/journal.pone.0165934)
Supplement: S4 Data — (PDF) [file pone.0165934.s005.pdf]

Neighbour Nets produced from the Great Language Game

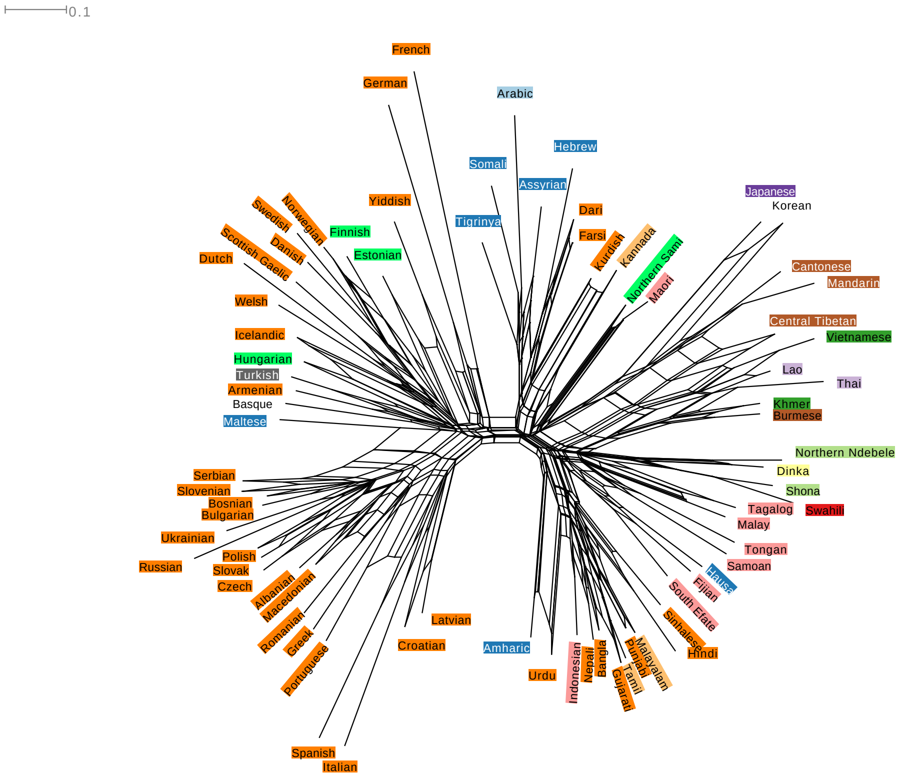

Figure 1: Neighbour net for responses from Africa

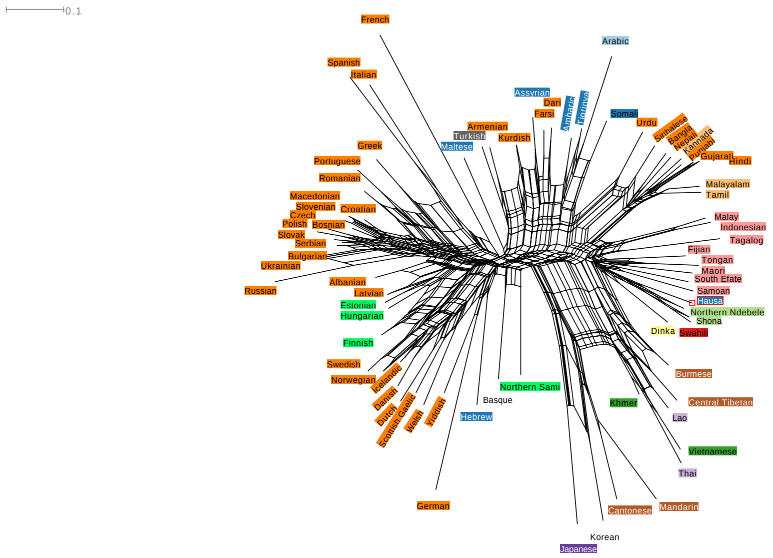

Figure 2: Neighbour net for responses from Asia

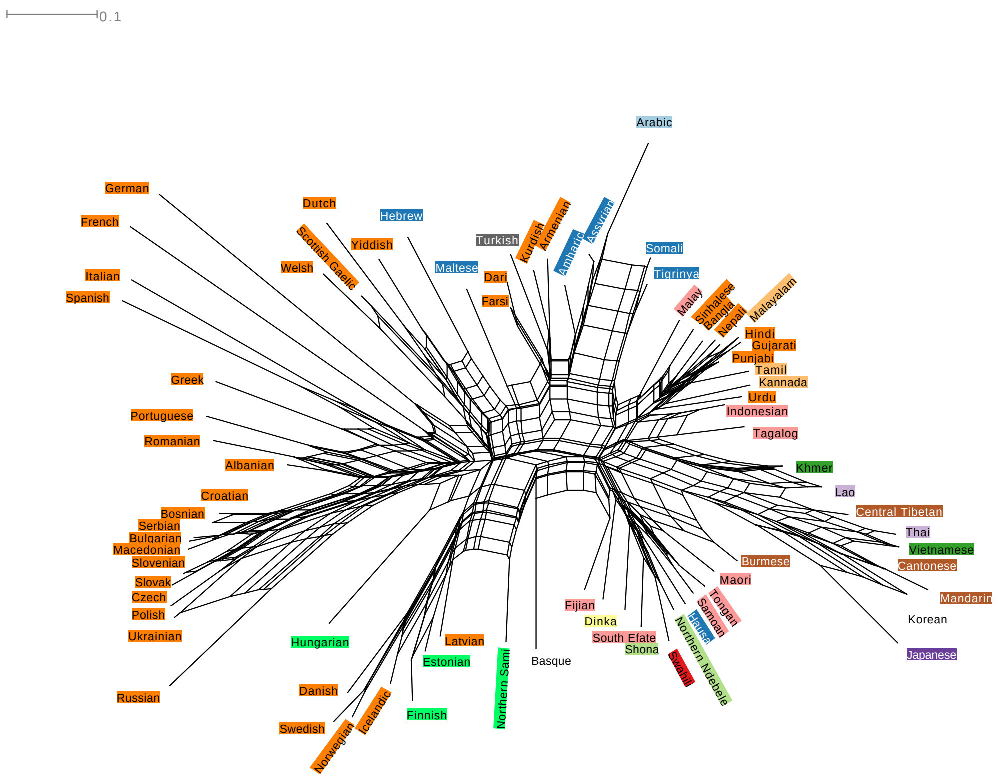

Figure 3: Neighbour net for responses from Europe

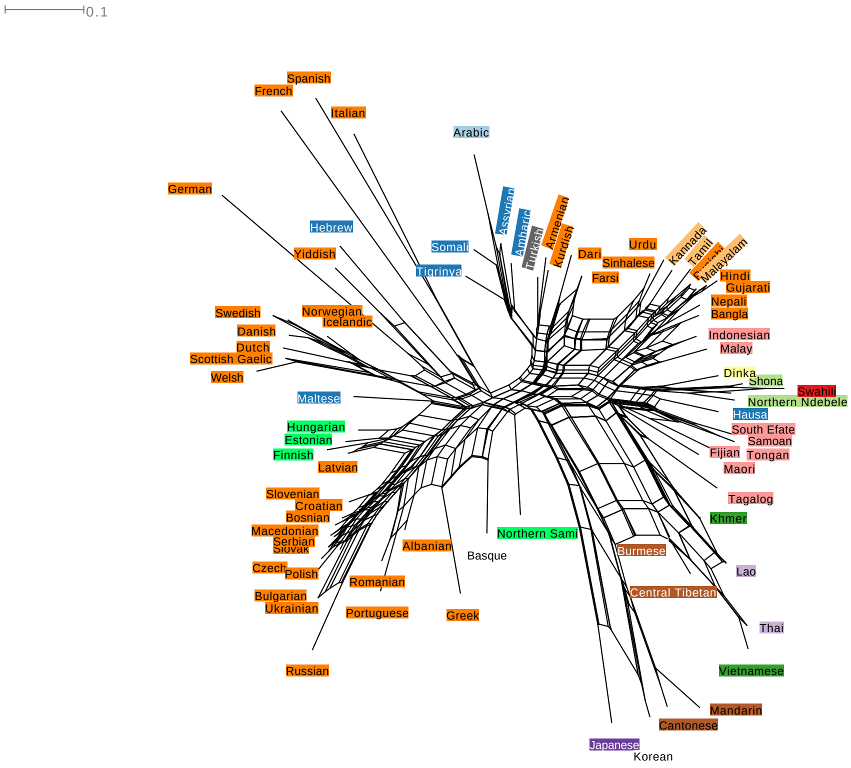

Figure 4: Neighbour net for responses from North America

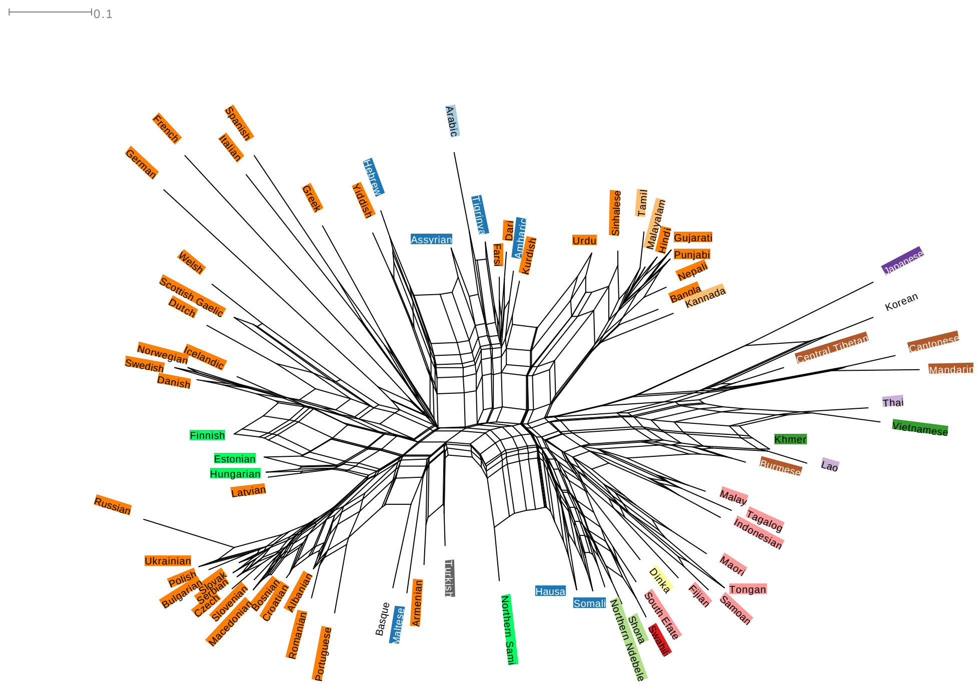

Figure 5: Neighbour net for responses from Oceania

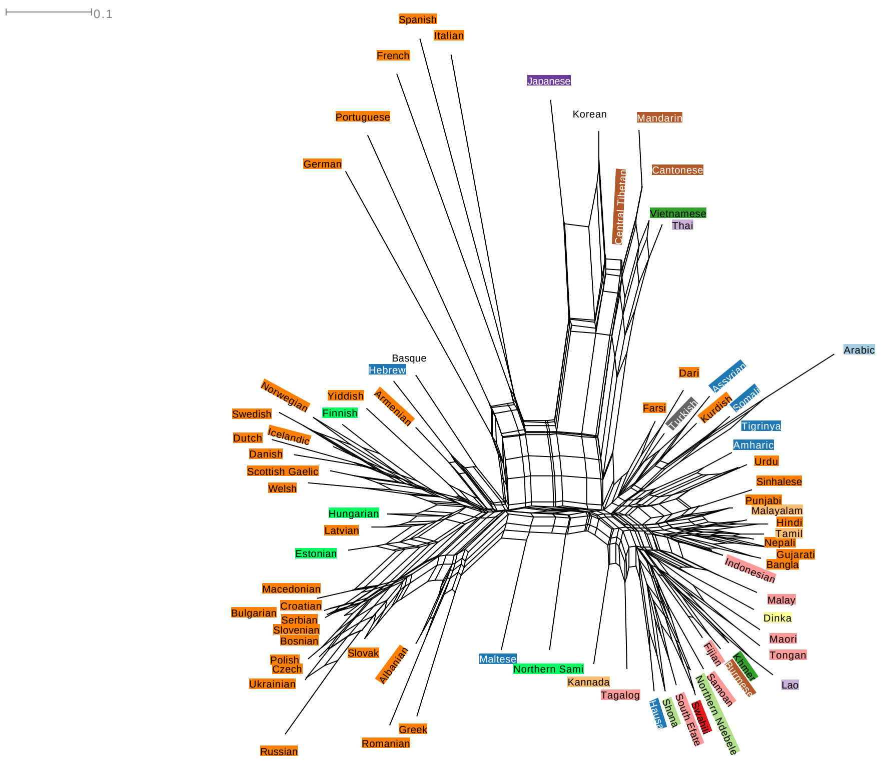

Figure 6: Neighbour net for responses from South America

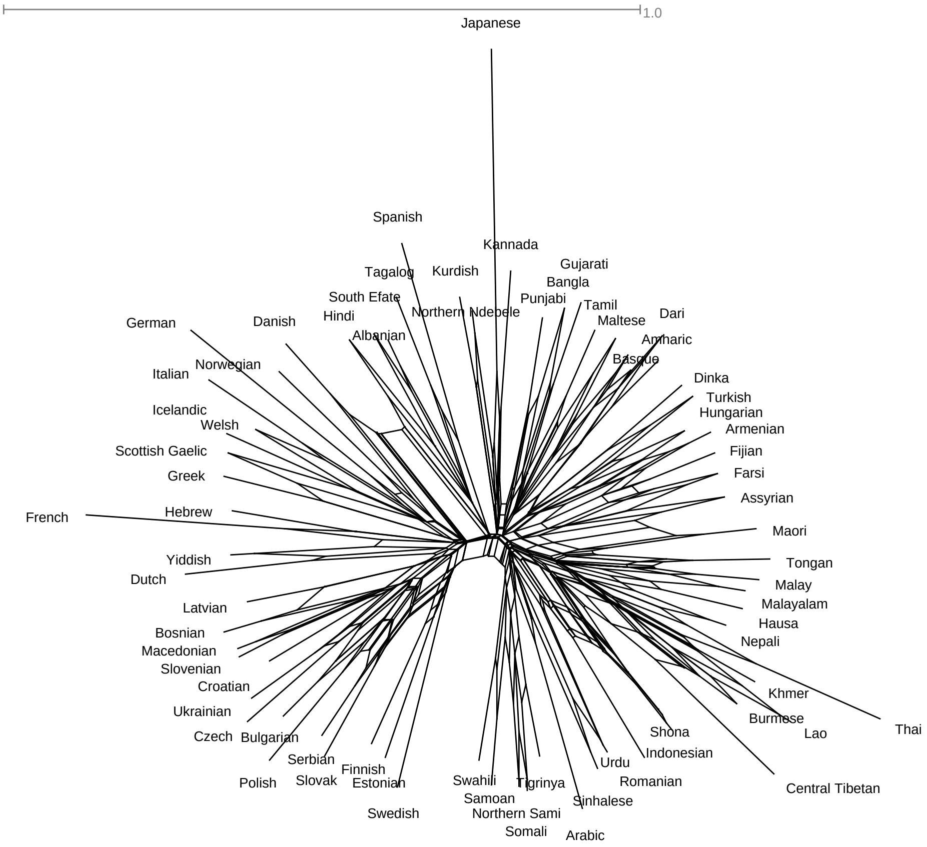

Figure 7: Neighbour net for responses from China

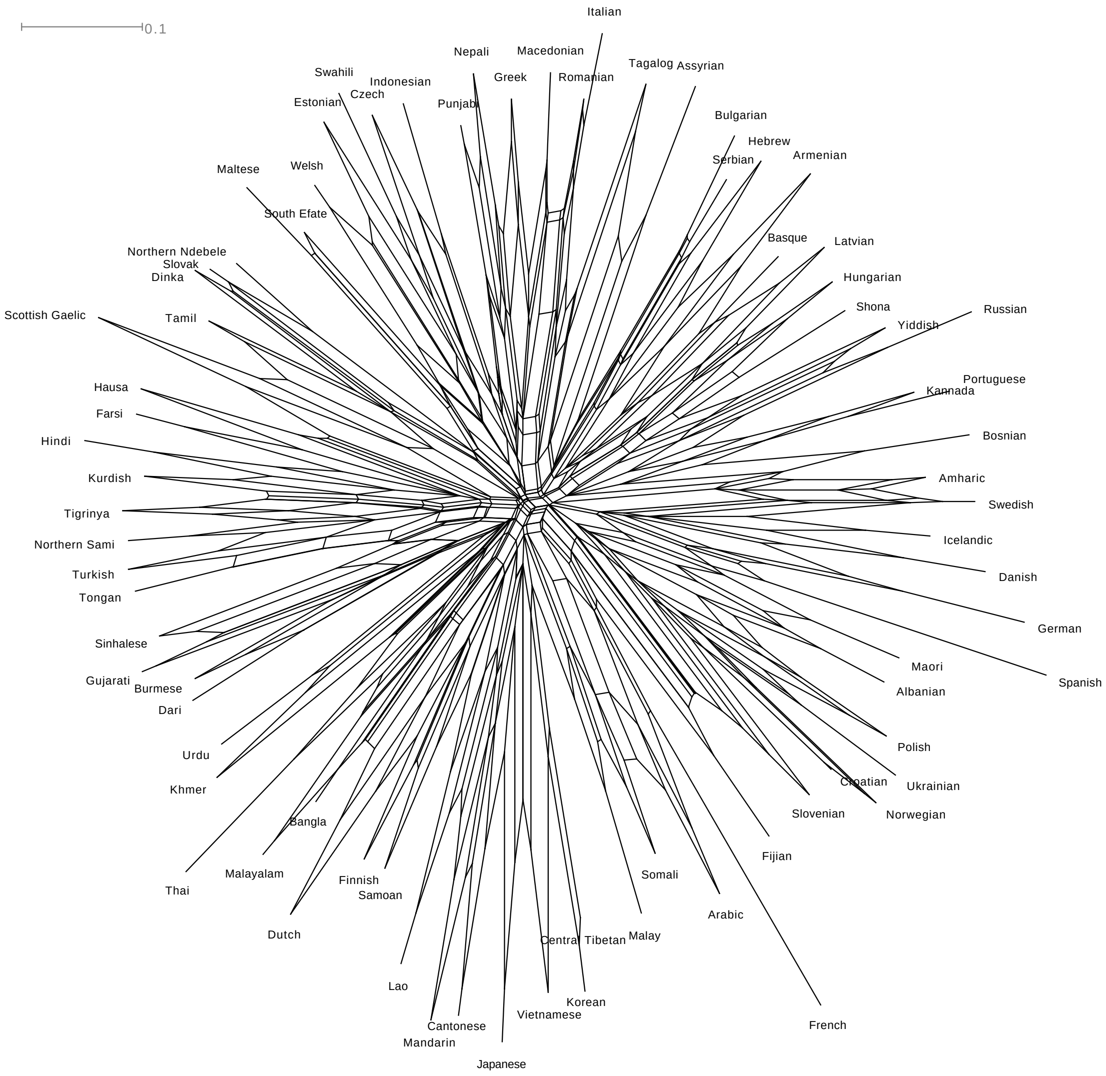

Figure 8: Neighbour net graph produced according to a Hebbian function. For each trial, the strength of the link between each guess and its target is increased, while the strength between the alternative candidates and the target is decreased. The amount of change is 1 / the number of candidates.

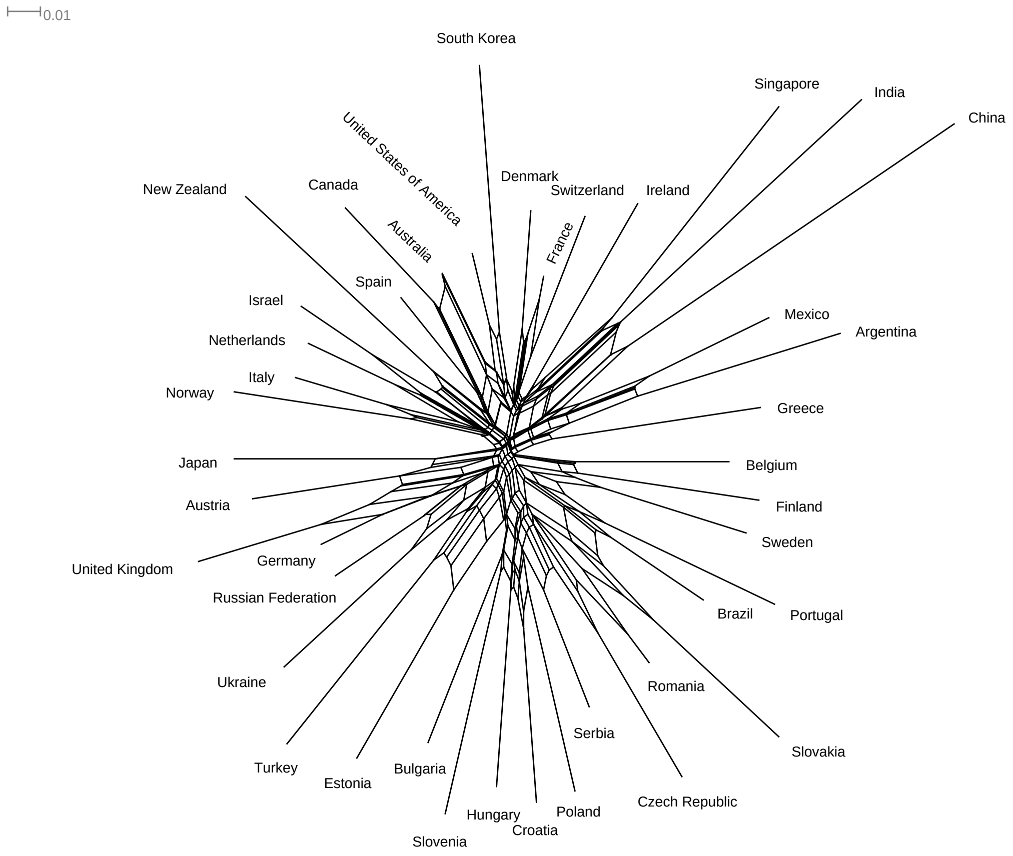

Figure 9: A confusion matrix which represents the differences in judgements between countries. A confusion matrix is made for each country, then a mantel test is conducted between each pair of confusion matrices to measure the differences between countries. This neighbour net is produced from that distance matrix of differences between countries. The delta score of the languages in this graph are correlated with the Greenberg diversity index (r = 0.31, p = 0.05, excluding two outliers- Singapore and India).

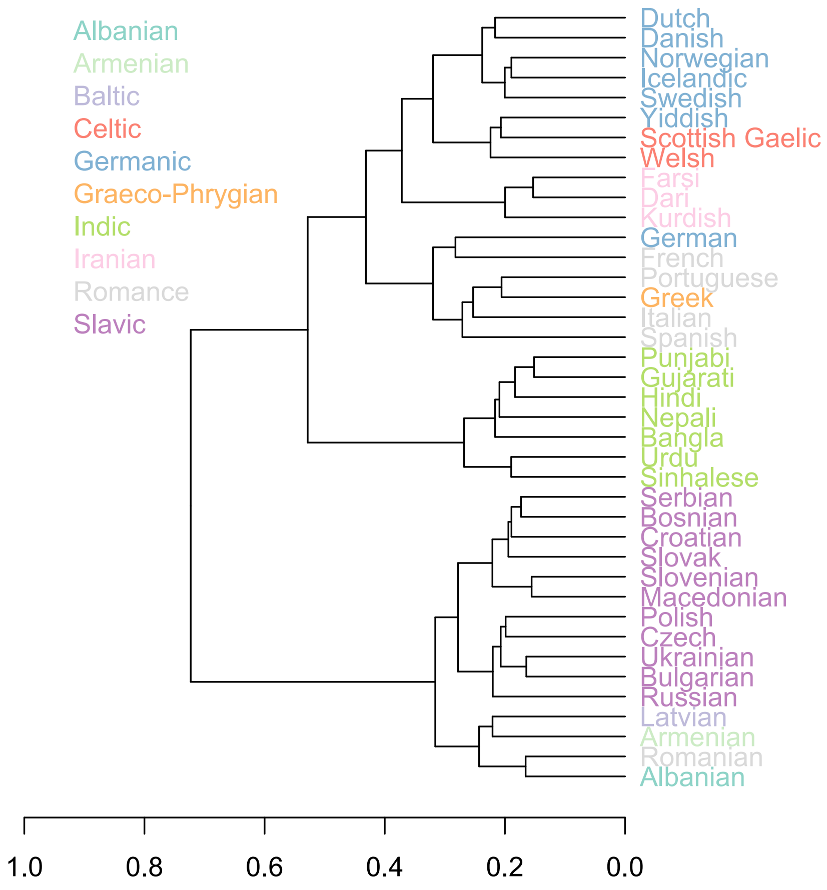

Figure 10: A binary tree constructed from the confusion matrix for Indo-European languages, according to Ward's method of hierarchical clustering. Languages are coloured according to their linguistic sub-group. The tree classifies some languages into sub-groups well (all Slavic languages belong to a single sub-branch, and the same is true for Indic, Iranian and Celtic sub-groups). All germanic languages except German and Yiddish are grouped together, and the Romance languages are also mostly grouped together. Interestingly, the cluster at the bottom of the graph groups Latvian, Armenian, Romanian and Albanian, and places Greek with the Romance languages.
